# Antioxidant and Wound Healing Bioactive Potential of Extracts Obtained from Bark and Needles of Softwood Species

**DOI:** 10.3390/antiox12071383

**Published:** 2023-07-04

**Authors:** Elisabeta-Irina Geana, Corina Teodora Ciucure, Radu Tamaian, Ioana Cristina Marinas, Diana Mădălina Gaboreanu, Miruna Stan, Carmen Lidia Chitescu

**Affiliations:** 1 National Research and Development Institute for Cryogenics and Isotopic Technologies, 240050 Ramnicu Valcea, Romania; corina.ciucure@icsi.ro; 2 Department of Microbiology and Biochemistry, Research Institute of the University of Bucharest-ICUB, 050567 Bucharest, Romania; gaboreanu.diana-madalina@s.bio.unibuc.ro (D.M.G.); miruna.stan@bio.unibuc.ro (M.S.); 3 National Institute of Research and Development for Biological Sciences, 060031 Bucharest, Romania; 4 Faculty of Medicine and Pharmacy, “Dunarea de Jos” University of Galati, 800008 Galati, Romania; carmen.chitescu@ugal.ro

**Keywords:** antioxidant activity, antimicrobial activity, anti-hemolytic activity, coniferous biomass, phytochemical bioactive compounds, polyphenols, PI3Kγ, protein-ligand docking

## Abstract

Interest in the extraction of phytochemical bioactive compounds, especially polyphenols from biomass, has recently increased due to their valuable biological potential as natural sources of antioxidants, which could be used in a wide range of applications, from foods and pharmaceuticals to green polymers and bio-based materials. The present research study aimed to provide a comprehensive chemical characterization of the phytochemical composition of forest biomass (bark and needles) of softwood species (*Picea abies* L., H. Karst., and *Abies alba* Mill.) and to investigate their in vitro antioxidant and antimicrobial activities to assess their potential in treating and healing infected chronic wounds. The DPPH radical-scavenging method and P–LD were used for a mechanistic explanation of the biomolecular effects of the investigated bioactive compounds. (+)-Catechin, epicatechin, rutin, myricetin, 4 hydroxybenzoic and p-cumaric acids, kaempherol, and apigenin were the main quantified polyphenols in coniferous biomass (in quantities around 100 µg/g). Also, numerous phenolic acids, flavonoids, stilbenes, terpenes, lignans, secoiridoids, and indanes with antioxidant, antimicrobial, anti-inflammatory, antihemolytic, and anti-carcinogenic potential were identified. The *Abies alba* needle extract was more toxic to microbial strains than the eukaryotic cells that provide its active wound healing principles. In this context, developing industrial upscaling strategies is imperative for the long-term success of biorefineries and incorporating them as part of a circular bio-economy.

## 1. Introduction

The promotion of renewable natural resources with potential applications in fields related to health, medicine, and nutrition is of great interest. The biomass of softwood species is rich in chemicals, with potential uses in various areas, from pharmaceutical and food industries to green polymers and bio-based materials [1,2]. The barks of softwood species contain high-level extractables, including a whole range of secondary metabolites such as valuable polyphenols, mostly condensed tannins, and hydrolysable tannins [3,4]. As natural antioxidants, polyphenols have important bioactive properties, which have been extensively described in the literature [5,6,7,8]. Several studies have confirmed that extractive substances from softwood bark show high antioxidant activity, revealing their potential for application in the pharmaceutical industry [9,10,11]. In addition, the use of polyphenolic extracts in various fields has also been studied, such as in alternative medicine (with topical applications for the wound healing process) [12,13], in the food industry to extend the product shelf life [14,15,16], in livestock farming as feed additives [17,18], and in the cosmetics industry [19,20]. Studies have also explored the use of polyphenolic extracts as adhesive resins [21] and for the removal of heavy metals [22].

Polyphenolic extracts from plant materials have been extensively used in wound healing (a process facilitated by four connected phases: hemostasis, inflammation, proliferation, and remodeling). Polyphenolic extracts assist in this process by producing physical, chemical, or mechanical agents based on a series of induced molecular and cellular events that aim to repair the defect in tissue integrity [23,24,25]. The wound healing process can be compromised and aggravated by pathogens and underlying pathologies (e.g.,: diabetes mellitus—DM). In acute injuries, a cascade of coagulation and vasoconstriction occurs at the tissue level to stop bleeding; this is followed by vasodilatation and the inflammatory response [26]. As wounds heal, proinflammatory macrophages facilitate fibroblast growth, collagen formation, and angiogenesis to induce healing, while cytokines and growth factors stimulate tissue re-epithelialization [27]. In contrast, chronic wounds (DM wounds, vascular wounds, and pressure ulcers) persist in a permanent inflammatory state whereby the levels of pro-inflammatory cytokines and proteases are increased [28]. These wounds may be based on a loss of cellular redox homeostasis due to increased levels of ROS and oxidative stress, which affect cellular components by degrading proteins, DNA, and lipids [29,30]. The cellular function could be restored by treatment with antioxidants such as phenolic compounds, which decrease ROS production and increase collagen expression [31]. Moreover, chronic wounds are easily infected, thus making healing difficult and potentially leading to life-threatening conditions [32]. Toxins, enzymes, adhesins, and other surface proteins associated with *Staphylococcus aureus* infections significantly disrupt host cells, potentially leading to necrosis, inflammation, and hemolysis [33]. On the other hand, the lipopolysaccharides (LPS) from Gram-negative bacteria (endotoxins) lead to chronic wounds [34]. Thus, chronic wound healing remains a major clinical challenge, and a mixture of agents with antimicrobial and antioxidant activity may act in concert to provide specific advantages in topical wound management [35]; this includes the treatment of wounds affected by biofilms produced by the ESKAPE pathogens (*Acinetobacter baumannii*, *Enterobacter* sp., *Enterococcus faecium*, *Klebsiella pneumoniae*, *Pseudomonas aeruginosa*, and *Staphylococcus aureus*) [13].

Conifers biomass contain valuable phytochemicals with therapeutic potential [36]. Thus, pine bark extracts have been reported to have several health benefits, including high antioxidant activity [37], cardiovascular protective properties [38], neuroprotective, anti-inflammatory, and anti-diabetic effects [37,39]. Oils extracted from *Cedrus deodara* (a member of the *Pinaceae* family abundantly found in coniferous forests) have good wound healing properties and anti-inflammatory effects [12]. Pine bark-based medication and food supplements are already commercially available (e.g., Pycnogenol and Enzogenol) [40,41].

Along with pine, spruce and fir are the most abundant conifer species in Eurasian forests. Among the species of spruce, *Picea abies* is endemic to the Romanian Carpathian area [42,43]. Highly bioactive compounds such as glycosylated monomeric stilbenes, astringin, taxifolin, piceid and isorhapontin [44,45], dimeric stilbenes with astringin and astringin-isorhapontin dimers [46], hydroxystilbenes resveratrol, isorhapontigenin, and piceatannol acetate derivatives have been identified in spruce bark and needles [47,48].

The recovery of phenolic compounds from forest waste biomass is gaining considerable attention [49,50,51], and the choice of the method and solvent used for extraction is a particularly important step [52]. Traditional extraction methods, such as maceration, Soxhlet extraction, and percolation, lead to low extraction yields, require relatively large quantities of solvents, and are often time-consuming [53]. Therefore, various extraction methods have been applied to increase phenolic extraction yield from wood bark, such as ultrasound-assisted extraction (UAE) [54,55], microwave-assisted extraction (MAE) [56], pressurized liquid extraction (PLE), supercritical fluid extraction [57,58], and enzymatic extraction [59].

The molecular targets for the effects of natural polyphenols are diverse and include enzymes, signaling pathways [60,61,62], and transcription factors [61,62]. Specifically, the phosphatidylinositol 3-kinase (PI3K) and protein kinase B (AKT) intracellular signal transduction pathway plays an important role in many biological responses. The PI3K/AKT signaling pathway triggers downstream effectors (protein kinases, lipid kinases, transcription factors, metabolic enzymes, regulators of small G proteins, and vesicle trafficking, etc.) and is crucial for growth and survival in response to the extracellular signals [63,64]. The PI3K/AKT signaling pathway downregulates various cellular processes, such as apoptosis, cell proliferation, the cell cycle, protein synthesis, glucose metabolism [63,65,66], and even telomere activity [67,68].

Regarding the skin, the PI3K/AKT signaling pathway is crucial for the survival, growth, proliferation, regeneration, and apoptosis of keratinocytes and dermal fibrocytes. Therefore, the PI3K/AKT signaling pathway is closely associated with epidermal barrier function, wound healing processes, and cellular senescence (skin aging). Therefore, it is supposed that fine-tuning the intensity of the PI3K/AKT signaling pathway may benefit skin homeostasis. Thus, understanding the precise regulation mechanisms of the PI3K/AKT signaling pathway is essential to advancing new therapeutic strategies to maintain skin homeostasis and wound healing processes [69].

After skin injury, the acute inflammatory response is activated by injury-associated molecular patterns (DAMPs), cellular Ca^2+^ waves, ROS release, lipid mediators, and chemokines. Necrotic and apoptotic cells release DAMPs that can activate various pathogen recognition receptors, such as Toll-like receptors (TLRs), on the surface of resident monocytes/macrophages, neutrophils, dendritic cells (DCs), T cells, mast cells, and keratinocytes. Keratinocytes play an important role in promoting the initiation of the inflammatory phase. They express different TLRs, which are upregulated in acute wounds [70]. The healing of chronic wounds is characterized by the upregulation of the NF-κB pathway, which involves an overexpression of pro-inflammatory cytokines and chemokines in keratinocytes. Impaired regulation of certain miRNAs in keratinocytes has been shown to delay wound healing. The chronic wounding process is also epigenetically regulated by miRNAs that control inflammatory responses by modulating signaling pathways, including Wnt/β-catenin, NF-κB, PI3K/Akt/mTOR, TGF-β/Smad, and VEGF [71].

According to Zulkefli et al. [72], it has been demonstrated that phenolic compounds, especially flavonoids, can modulate the signaling pathways involved in wound healing, e.g., Wnt/β-catenin, TGF- β, Notch, Bone morphogenetic protein (BMP) pathway, Hedgehog, bone morphogenetic protein (BMP), Wnt signaling, PI3K/AKT/mTOR, Notch, AKT, MAPK signaling pathways, ERK, P38, JNK, and Akt. The three highly homologous isoforms of the AKT serine/threonine kinase family (AKT1, AKT2, and AKT3) represents the vital signaling center of the PI3K/AKT signaling pathway; however, AKT’s activation is dependent on PI3K, a large family of evolutionarily conserved lipid kinases [66]. Some extractable plant secondary metabolites (phenols), like quercetin and its analogs, have already been classified as broad-range inhibitors of protein kinases, and the binding patterns of quercetin and myricetin with the class IB phosphoinositide 3-kinase PI3K-gamma (PI3Kγ) were solved via X-ray crystallography [73] and described by the authors of The Research Collaboratory for Structural Bioinformatics Protein Data Bank (RCSB PDB) [74,75,76]. The catalytic subunit of PI3Kγ (p110γ, encoded by the PIK3CG gene) pairs either with the p101 adaptor subunit (encoded by the PIK3R5 gene) or the p84 adaptor subunit (encoded by the PIK3R6 gene), two non-homologues adaptors to the two p85 adaptor subunits (p85α, encoded by the PIK3R1 gene, and p85β, encoded by the PIK3R2 gene—common to the class IA PIK3s) or any other proteins [66,77]. PI3Kγ has been identified as a strategic target in the treatment of chronic inflammation and auto-immune diseases [66,77], cancer [66,73,77,78,79], and obesity-related disorders [66,77], as well as in wound healing [80,81]. Moreover, PI3Kγ is involved in diabetes mellitus (DM) pathology and its ensuing clinical complications (e.g., the healing process of chronic DM wounds) [73,82].

Protein—Ligand Docking (P–LD) is a molecular docking technique (in silico technique) used to predict the position and orientation of a ligand (a low-molecular weight organic compound) when it is bound to an enzyme or a protein receptor. The purpose of P–LD is to identify the most energetically favorable binding mode (pose) between the protein and ligand and it is currently used not only in drug discovery but also for the exploration of the biomolecular mechanisms of action of natural bioactive compounds [83]. P–LD can be used to discover high-quality lead molecules, narrow down the range of molecules that need to be tested in vitro and in vivo, reduce the economic investment in preliminary testing, and minimize the cost associated with failure in the later stages of the development of new therapeutic molecules and new biomaterial-based medical devices. The strategy of combining various wet-lab and in silico techniques for exploring the mechanisms of action of natural phenolics and their synthetic analogs has been used previously in many exploratory studies, including the combination of the DPPH radical-scavenging method and P–LD [84,85,86]. Previously, the designated target enzyme (PI3Kγ) was investigated by using its RCSB PDB entry 1E90 (resolution = 2.70 Å) [73] against a set of major compounds identified in the essential oils and lipophilic extract of *Moricandia sinaica* Boiss [84].

The present research study aimed to characterize the phytochemical bioactive composition of the forest biomass (bark and needles) of softwood species such as spruce (*Picea abies* L., H. Karst.) and fir (*Abies alba* Mill.) in order to identify the specific phytochemical biomarkers of each type of biomass. Antimicrobial activity was assessed using reference and clinical strains isolated from wound infection, and anti-hemolytic activity was assessed by using AAPH (2,2′-Azobis(2-amidinopropane) dihydrochloride) to induce the hemolysis of red blood cells (RBCs). This enabled us to evaluate the wound healing potential of the extracts. The combined DPPH radical-scavenging method and P–LD for the most abundant compounds were performed to evaluate the potential wound healing effect of the plant extracts by modulating the PI3K/AKT signaling pathway using 1E8W RCSB PDB entry for PI3Kγ.

## 2. Materials and Methods

### 2.1. Chemicals

All the chemicals used were of analytical grade. For extraction, absolute anhydrous ethanol was supplied by Merck (Darmstadt, Germany), while deionized water was produced by using a Milli-Q Millipore system (Bedford, MA, USA). For spectrophotometric assays, Folin–Ciocalteu phenol reagent (2 N), radical scavenging assay reagents DPPH, and 6-hydroxy-2,5,7,8-tetramethyl-2-carboxylic acid (Trolox) were purchased from Sigma-Aldrich (St. Louis, MO, USA), and anhydrous sodium carbonate, aluminium chloride, and sodium acetate were purchased from Merck (Darmstadt, Germany). To facilitate UHPLC–MS analysis, LC–MS-grade acetonitrile and water were purchased from Merck (Darmstadt, Germany), and formic acid was purchased from Fisher Scientific (Loughborough, UK).

The following reference standards of phenolic compounds were purchased from Sigma-Aldrich (Steinheim, Germany): phenolic acid (chlorogenic, vanillic, caffeic, syringic, p-hydroxybenzoic, gallic, p-coumaric, ferulic, and cinnamic), flavonoids ((+)-catechin, (−)-epicatechin, quercetin, apigenin, galangin, myricetin, rutin, naringin, kaempferol, isorhamnetin, chrysin, pinocembrin, pinostrobin), and stilbenoid trans-resveratrol. The stock solutions of phenolic acids with trans-resveratrol and flavonoids were prepared in methanol. The working standards were made by diluting the stock solutions with water/methanol in an 80:20 (*v*/*v*) ratio. Both the stock and working standards were stored at 4 °C until further use.

### 2.2. Forest Biomass Origin and Preparation

The spruce (*Picea abies* L., H. Karst.) and fir (*Abies alba* Mill.) barks and needles were collected in October 2020 from 27 to 32 years old trees located within the Voineasa (average altitude: 700 m) and Malaia (average altitude: 1130 m) localities of Southern Carpathians, Romania. The trees had diameters that ranged from 31 to 35.5 cm. We collected 13 of each spruce bark and needles and 4 of each fir bark and needles (from trees located at different altitudes). The raw plant materials were dried at 40 °C using an Biovita drier (Biovita, România), ground in a two-knife mill (Fritsch, Idar-Oberstein, Germany), and then ground into a fine powder by using a Retsch 200 mill (Haan, Germany). The bark was stored in sealed bags at +4 °C until they were required for use in the experiments.

### 2.3. Microwave-Assisted Extraction (MAE) of Bioactive Compounds from Bark and Needles

Microwave-assisted extraction was performed with a CEM Mars 6 closed microwave system (CEM Corp., Matthews, NC, USA)), with adjustable power settings ranging from 100 to 1600 W, closed vessel reactions (GreenChem, 100 mL), power and temperature sensors, and steering and cooling systems. To determine the best extraction solvent, different extractions were carried out at 300 W for 5 min with ethanol and methanol (30%, 50%, and 80%) solutions at 1:30 liquid/solid ratio (g/g). Low-power extraction (300 W) was used to better manage the reaction of solvents in a closed system [87]. The optimal extraction conditions that allowed us to obtain a high total polyphenol content (mg GAE/g) were as follows: 50% ethanol extraction solvent, 10 min ramp to 50 °C, followed by 5 min hold. Briefly, 1.0 g of bark/needle powder was weighed into extraction vessels, and then 30 mL of 50% ethanol was added. Teflon stir bars were inserted, and the vessels were closed and subjected to the MAE program. At the end of the extraction process, the extraction vessels were allowed to cool for 30 min at room temperature and then the extracts were centrifuged for 15 min at 4 °C and 10,000 rpm (Hettich Rotina 380 R Centrifuge, Tuttingen, Germany). After filtration through a 0.45 mm membrane, the aliquots of samples were preserved at −4 °C until they were required for analysis. Two replications were carried out for each treatment. The remaining extracts were evaporated to remove the ethanol in a gentle stream of nitrogen using a TurboVap LV (Biotage, Uppsala, Sweden) and then lyophilized using a Labconco freeze dryer (Biotage, Uppsala, Sweden). The obtained final masses were weighted to calculate the Total Extraction Yield, with the obtained values being 5.8% and 14.0% for fir and spruce bark, respectively, and 4.5% and 7.5% for fir and spruce needles, respectively.

### 2.4. Bioactive Polyphenolic Characterization of Bark and Needles Extracts

#### 2.4.1. Bioactive Characteristics by UV-Vis Spectrophotometric Methods

The bioactive characteristics (total polyphenols (TP), total flavonoids (TF) and antioxidant activity (AA)) of the bark and needle extracts were determined by UV-Vis spectrophotometric measurements taken using an Specord 250 Plus UV-Vis spectrophotometer (Analytic Jena, Jena, Germany) equipped with 1 cm pathlength quartz cells. All analytical determinations were conducted in duplicate, and results were averaged.

The Total polyphenols (TP) were determined by using the Folin–Ciocalteau method according to the protocol described by Ciucure et al. [88]. In brief, 100 μL of 5-fold diluted extract was added to test tubes and mixed with 5 mL ultrapure water and 200 µL Folin–Ciocalteau reagent. After 5 min of reaction at room temperature and protected from light, 300 µL of 20% sodium carbonate solution was added to stop the reaction and to generate a characteristic blue color for 2 h. The resulting blue color had a maximum absorbance peak of 675 nm, with the absorbance being proportional to the amount of phenolic compounds. Water was used as blank. A calibration curve was built using gallic acid as a standard (0–1250 mg/L) to quantify the samples. The results were expressed as mg gallic acid equivalent (GAE) per gram of bark (mg GAE/g bark).

The Total Flavonoids (TF) were determined by using the AlCl_3_ method as described by Geana et al. [89]. Briefly, 0.5 mL of extract were treated with 0.4 mL of 25 g/L AlCl3 solution, 0.5 mL of 100 g/L CH3COONa solution and 4 mL distilled water. After 15 min, the absorbance of the mixture was measured at 430 nm against water as blank. Total flavonoid content (TF) was quantified as mg quercetin equivalent (QE) per gram of bark using calibration curve obtained in the concentration range of 0–125 mg/L quercetin.

Antioxidant activity (AA) was determined by the DPPH (2,2-diphenyl-1-picrylhydrazyl) radical-scavenging method, as described by Geana et al. [89]. In this assay, after being left to stand for 20 min at room temperature, 6 mL of 0.09 mg/mL DPPH methanolic solution was mixed with 0.5 mL aliquots of 5-fold diluted extract, and the absorbance was measured at 517 nm against methanol as blank. Absorbance measurements were transformed to antioxidant activity using Trolox as standard, (in the concentration range of 50–1000 µmol/L), and the antioxidant activity was expressed as µmol/L Trolox equivalents per gram of bark/needles (dw).

#### 2.4.2. Target and Non-Target Polyphenolic Composition by HRMS Analysis

Chromatographic determinations were carried out using a high-resolution Q Exactive mass spectrometer Focus Hybrid Quadrupole—OrbiTrap (Thermo Fisher Scientific, Bremen, Germany) equipped with HESI and coupled to a high-performance liquid chromatograph UltiMate 3000 UHPLC (ThermoFisher Scientific, Bremen, Germany) with a DAD detector set at 280 nm. Separations were performed on Kinetex^®^ C18 column (100 × 2.1 mm, 1.7 µm particle diameter) at 30 °C. The mobile phase consisted of: solvent A—water with 0.1% formic acid and solvent B—acetonitrile with 0.1% formic acid. The UHPLC gradient for mass screening was: 0 min, 10% B, 0–10 min, 20% B; 10–20 min, 30% B; 20–30 min, 50% B; 30–33 min, 70% B; 33–43 min, 90% B; 43–45 min, 10%B and equilibration of the column for the next run. The flow rate was 0.3 mL/min, and the injection volume was 10 μL. All samples were filtered with 0.45 μm PTFE membrane filter prior to injection. The mass spectrometer was operated in negative mode in a range between 100 and 1000 *m*/*z* at a resolution of 70,000. HESI-source parameters were as follows: spray voltage—2.8 kV, capillary temperature—320 °C, auxiliary gas heater temperature—413 °C, sheath and auxiliary gas flow (N_2_)—35 and 10 (arbitrary units), respectively. Polyphenolic compounds from the samples were identified and quantified according to their spectral characteristics (mass spectra, accurate mass, and characteristic retention time) and compared against external standard solutions analyzed under the same conditions. In order to confirm the identified compounds, fragmentation studies were performed by employing a data-dependent scan with collision-induced dissociation (CID). The normalized collision energy of the CID cell was set at 25, 35, and 45 eV. Xcalibur software (Version 4.1) was used for instrument control, data acquisition, and data analysis. For quantification, in a concentration range between 0 and 2000 μg/L for each of the phenolic acids and flavonoids, calibration was performed via serial dilution with methanol of the standard mixture of concentration 10 mg/L. All stock and working solutions were stored in the dark at 4 °C. Calibration curves based on duplicate injections revealed good linearity, with correlation coefficients (R2) values exceeding 0.99 (peak areas versus concentration). For the non-target UHPLC-MS/MS screening, data processing, analysis, and interpretation were performed via Compound Discoverer v. 2.1 (Thermo Scientific, Waltham, MA, USA) software using an untargeted metabolomics working template combined with reference library databases containing accurate MS data, such as ChemSpider “www.chemspider.com (accessed on 10 May 2023)” and PubChem “https://pubchem.ncbi.nlm.nih.gov/ (accessed on 15 May 2023)”, and LC-MS spectral databases such as Mass Bank “www.massbank.eu (accessed on 10 May 2023)” and Wiley Science Solutions “www.spectrabase.com (accessed on 15 May 2023)”.

### 2.5. Antimicrobial Activity of Bark and Needles Extracts

#### 2.5.1. Microbial Strains

Antimicrobial activity was tested on Gram-positive (*Staphylococcus aureus* sc pl, multi-drug-resistant clinical isolates of *S. aureus* (MRSA), *Enterococcus faecalis* ATCC 19433) and Gram-negative (*Escherichia coli* C10, *Pseudomonas aeruginosa* ATCC 27853, *Serratia marcescens* 5c5k) bacterial strains, as well as yeast (*Candida albicans* ATCC 10231). The clinical strains were obtained from the Microbial Strain Collection of Faculty of Biology, University of Bucharest, Romania, isolated from infected wounds, and confirmed via MALDI-TOF.

#### 2.5.2. Qualitative Antimicrobial Activity

Antimicrobial activity was determined by employing an adapted version of the diffusion method described by Marinas et al. [90]. Briefly, a microbial inoculum with a density corresponding to 0.5 McFarland standard for bacterial strains and 1 McFarland for yeast strain was evenly swabbed on the agar surface, and thereafter 10 µL of the sample was spotted on the solid medium. The results were analyzed in triplicate and are expressed as the mean of diameter zone inhibition (DIZ) ± standard deviation (SD). In the same conditions, ethanol 50% was used as solvent control.

#### 2.5.3. Quantitative Antimicrobial Activity

Quantitative analysis was performed by using a binary serial microdilution method in liquid medium (Brain Hart Infusion for bacteria and Sabouraud for yeast) in a 96-well plate. The concentration range for alcoholic extracts was from 7.81 to 500 µL/mL. Simultaneously, serial dilutions were made with 50% ethanol under the same working conditions in order to obtain a negative control. Each well was inoculated with 10 μL microbial suspension, adjusted to 1.5 × 10^8^ CFU/mL from 18 to 24 h grown cultures. The MIC values were established both macroscopically, as the last concentration at which no microbial growth was observed, and spectrophotometrically. The absorbance of the microbial cultures was measured at 620 nm by using the FlexStation 3 UV-Vis (Molecular Devices, San Jose, CA, USA) Spectrophotometer.

#### 2.5.4. Prevention of Biofilm Formation

Following the quantitative analysis of the antimicrobial activity, the adherence of biofilm biomass was assessed after fixation with methanol and crystal violet staining (0.1%) by using the slime method. The absorbance of the biological material resuspended in 33% acetic acid was measured at 490 nm.

### 2.6. Biocompatibility

Cytotoxicity was tested on HaCaT non-tumor human keratinocytes. The cell line was cultured in DMEM medium (Dulbecco’s Modified Eagle Medium, Sigma-Aldrich) supplemented with 10% fetal bovine serum (Sigma-Aldrich) and 1% Pen/Strep (penicillin/streptomycin solution, 50 µg/mL—Sigma-Aldrich) at 37 °C, 95% humidity, and 5% CO_2_. Cells were washed with phosphate-buffered saline (PBS, Sigma Aldrich), trypsinized (0.25% Trypsin-0.53 mM EDTA, Thermo Scientific), and counted using Trypan Blue and a Burker–Turk counting chamber. Different concentrations of extracts (5–50 µL/mL) were incubated with the cells (which were 24 h pre-seeded at a density of 2 × 10^4^ cells/well) in a final volume of 200 µL growth medium for 24 h (37 °C, 95% humidity, 5% CO_2_).

#### 2.6.1. MTT Assay

The MTT assay was used to assess cell viability and proliferation in the presence of extracts. This viability test facilitates the quantitative evaluation of live cells in culture. The compound MTT [3-(4,5-dimethylthiazol-2-yl)-2,5-diphenyltetrazolium] is permeable to living cell membranes and metabolized into soluble formazan crystals. The cells were incubated for 2 h with MTT at 37 °C, 95% humidity, 5% CO_2_. The formed formazan was dissolved in isopropanol, and the absorbance was measured at 595 nm on a FlexStation 3 plate reader (Molecular Devices, San Jose, CA, USA). The data obtained was analyzed using log (inhibitor) vs. response—Variable slope (four parameters) analysis function with the help of Prism GraphPad 9.0 software.

#### 2.6.2. Lactate Dehydrogenase (LDH) Release Assay

The culture medium was collected after 24 h of incubation with the extracts, and LDH release was measured using the Cytotoxicity Detection Kit PLUS (Roche, NY, USA) according to the manufacturer’s instructions. Volumes of 50 µL culture supernatants were mixed with 50 µL reaction mixture of catalyst and dye solution and incubated for 30 min in dark conditions. The reaction was stopped by adding 50 µL of stop solution, and the absorbance was measured at 490 nm using a microplate reader (Flex Station 3, Molecular Devices, San Jose, CA, USA).

### 2.7. Hemocompatibility

#### 2.7.1. Hemolytic Index

The hemolytic index of the extracts was measured by using a spectrophotometric procedure as described elsewhere [91]. Nine milliliters of ram blood were collected in acid citrate dextrose (ACD) tubes and centrifuged for 10 min at 2000 rpm. The supernatant was removed, and the pellet was washed thrice with PBS (0.2 M, pH 7.4) before re-suspension in sterile saline solution (0.9%). Furthermore, 50 µL of the extracts (50–500 μL/mL in PBS, pH = 7.4) was dispensed into 200 µL of erythrocyte suspension (10%). The mixtures were incubated 1 h at 37 °C. The samples were centrifuged at 2000 rpm for 10 min, and the absorbance of the supernatant was measured at 540 nm. Relative hemolysis was assessed in comparison with the hemolysis in deionized water (A+), which was set as 100%. For the negative control, phosphate-buffered saline was used (A−). Each set of experiments was performed in triplicate, and inhibitory activity was calculated; expressed herein as percent inhibition of hemolysis. The hemolysis (%) was calculated as follows:

Hemolysis (%) = (A sample − A−) × 100/(A+ − A−)

#### 2.7.2. Anti-Hemolytic Activity

The anti-hemolytic potential of the extracts was measured via the use of a previously described spectrophotometric procedure [92] (with some modifications) [93,94]. Nine milliliters of ram blood were collected in acid citrate dextrose (ACD) tubes and centrifuged for 10 min at 2000 rpm. The supernatant was removed, and the pellet was washed thrice with PBS (0.2 M, pH 7.4) before re-suspension in saline solution (0.9%). Furthermore, 50 µL of the extracts (35–200 μL/mL in PBS, pH = 7.4) was dispensed into 200 µL of erythrocyte suspension (10%). The mixtures were incubated for 20 min at 37 °C, and 100 µL of AAPH (200 mM final concentration) was added to the reaction mixture to induce the oxidative degradation of the membrane lipids. The mixtures were incubated for 4 h at 37 °C. Subsequently, the samples were centrifuged at 2000 rpm for 10 min, and the absorbance of supernatant measured at 540 nm. The relative hemolysis was assessed in comparison with the hemolysis in the AAPH-treated negative control (A AAPH), which was set as 100%. For the positive control, phosphate-buffered saline instead of AAPH was used (A PBS). Each set of experiments was performed in triplicate, and inhibitory activity was calculated; expressed herein as percent inhibition of hemolysis. A stock solution of ascorbic acid (1 mg/mL) was used treated in a similar manner to the method employed for the samples in a range of concentrations between 35 and 200 μL/mL. The hemolysis (%) was calculated as follows:

Hemolysis (%) = (A sample − A PBS) × 100/(A AAPH − A PBS)

### 2.8. Statistical Analysis

The data are expressed as means ± standard deviation (SD) (determined via triplicate analysis). The statistical analysis was conducted using GraphPad Prism 9v. The data were analyzed using ordinary two-way ANOVA with two-stage step-up for multiple comparisons (Benjamini, Kriefer, and Yekutieli) for biological assays. The level of significance was set to *p* < 0.05. Principal component analysis (PCA) and Heat Map Analysis (HMA) were carried out on the data matrix, including for the rows corresponding to the analyzed extracts, and variables derived from both the target and non-target HRMS analyses were analyzed by using Microsoft Excel 2010 (Microsoft, Redmond, WA, USA) and XLSTAT Add in soft version 15.5.03.3707 (Addinsoft, New York, NY, USA).

### 2.9. Protein—Ligand Docking (P–LD)

A molecular docking study was carried out for several identified phytochemicals to evaluate the potential wound healing effect of the plant extracts (which is facilitated by modulating the PI3k/Akt signaling pathway and using 1E8W RCSB PDB entry for PI3Kγ due its better resolution; 2.50 Å) [73]. Two comparative P–LD runs were performed against the selected molecular target by using two different software types with two different docking algorithms, namely, (1) AutoDock Vina v.1.2.0 algorithm [95,96] with PyRx—Python Prescription v.0.9.7 software [97] as user interface (UI) control and (2) SwissDock web-service “http://www.swissdock.ch/ (30 May 2023)” [98] with EADock DSS algorithm [98,99]. A detailed description of the molecular docking technique (in silico technique) of each P-LD runs is provided in the Supplementary Materials. The co-crystallized ligand of PI3Kγ/1E8W (quercetin) was re-docked, serving as a reference or control molecule for both P–LD runs.

## 3. Results and Discussions

### 3.1. Bioactive Characteristics

**Figure 1:**
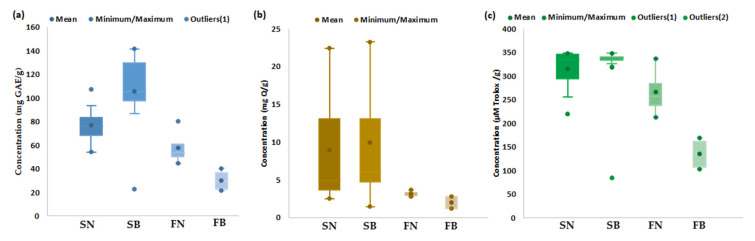
Bioactive characteristics of coniferous biomass (spruce needles—SN (*n* = 13), spruce bark—SB (*n* = 13), fir needles—FN (*n* = 4), and fir bark—FB (*n* = 4)): (**a**) Total polyphenols (TP), (**b**) Total flavonoids (TF), and (**c**) Antioxidant activity (AA).

Quantitative estimation of the bioactive characteristics (TP, TF, AA) of spruce and fir biomass (bark and needles) collected from mature trees distributed at different altitudes was performed in order to generate an overall estimation of the bioactive characteristics of each type of biomass. The obtained results are presented in a range of variation, mean, minimum, and maximum values, as shown in Figure 1 and Table S1.

As highlighted in Figure 1, spruce bark and needles seemingly have more bioactive characteristics, with TP values (Figure 1a) ranging between 22.92 and 141.53 mg GAE/g for bark and between 54.31 and 107.06 mg GAE/g for needles, with average values of 105.83 mg GAE/g for bark and 77.03 mg GAE/g for needles. The amount of TP in the fir bark and needles ranged between 21.64 and 40.28 mg GAE/g for bark and between 44.50 and 80.37 mg GAE/g for needles, with average values of 30.21 mg GAE/g for bark and 58.00 mg GAE/g for needles. The literature data indicated similar values of TP in *Picea abies* bark (11.03 mg GAE/g [57], 13.30 mg GAE/g [100], 54.97 mg GAE/g [101], 130.26 mg GAE/g [58]), higher values in *Picea mariana* bark (404.0 mg GAE/g [102], 426.0 mg GAE/g) [103], and lower values of TP in fir bark (14.32 mg GAE/g) [55]. No data were available in the literature for the TP content of spruce and fir needles, but the quantified values were lower compared to those reported for pine needles (274.38 mg GAE/g) (see Table S2).

The amount of TF (Figure 1b) ranged between 1.46 and 23.24 mg Q/g for bark and between 2.53 and 22.43 mg Q/g for needles, with average values of 9.91 mg Q/g for bark and 8.94 mg Q/g for needles. The amount of TF in fir bark and needles ranged between 1.24 and 2.81 mg Q/g for bark and between 2.76 and 3.67 mg Q/g for needles, with average values of 2.01 mg Q/g for bark and 3.17 mg Q/g for needles. The literature data (Table S2) reported the following values for TF (expressed as quercetin equivalents): 3.54–53.4 mg Q/g for spruce bark [58,100,101,102], 7.46 mg Q/g for fir bark [100], 8.29–81.9 mg Q/g for pine bark [49,100], and 1.6 mg Q/g for pine needles [104].

Antioxidant activity of the studied coniferous biomass (Figure 1c) ranged between 84.77 and 348.51 µMoli Trolox/g, dw and from 220.26 to 348.76 µmol Trolox/g, dw for spruce bark and needles, respectively (Table S1), with average values of 318.59 µmol Trolox/g for bark and 316.12 µmol Trolox/g for needles. Lower values of AA were observed for fir bark (103.26–170.01, average value of 135.77 µmol Trolox/g) and fir needles (212.99–337.19, average value of 265.91 µmol Trolox/g). The literature data reported higher values of AA in spruce (308.00 µmol Trolox/g [103] and 404.12 µmol Trolox/g [100]) and fir (269.55 µmol Trolox/g [100]) barks (Table S1). It should be mentioned that, in the literature data, the bioactive properties were also reported as mg GAE/g extract for TP and mg Q or mg CE/g extract for TF and µg TE/mL extract (see Table S1).

### 3.2. Quantification of Polyphenolic Compounds in Picea abies L., H. Karst., and Abies alba Mill. Barks and Needles

A number of 24 compounds, including 11 phenolic acids and derivatives, 12 flavonoids, and stilbene t-resveratrol, were unambiguously identified and quantified in the coniferous biomass based on a comparison with available reference standards (retention time, high-resolution accurate mass, and fragmentation pattern) (Table S3). The quantitative results of the individual phenolic compounds in coniferous biomass (bark and needles of *Picea abies* L., H. Karst., and *Abies alba* Mill.) are presented in Table 1.

Flavonoids represent the most dominant bioactive compounds in spruce and fir biomass (bark and needles), and (+)-catechin is the representative compound of coniferous biomass, with values ranging between 108.7 and 1529.4 µg/g in spruce needles, 48.5–1420.4 µg/g in spruce bark, 94.0–894.5 µg/g in fir needles, and 53.8–186.6 µg/g in fir bark. The obtained data were comparable with data reported in the literature for fir (1499.61 µg/g) and pine (1502.3 µg/g) needles [100]. It should be noted that flavonoids such as (−)-epicatechin, myricetin, quercetin, isorhamnetin, and apigenin were also quantified in important amounts in both spruce and fir bark and needles; generally, higher amounts corresponded to spruce bark and needles rather than fir bark and needles. Higher amounts of kaempferol and rutin were quantified in spruce and fir needles, while lower amounts correspond to spruce and fir bark. Pinocembrin, chrysin, and galangin were quantified in higher amounts in fir biomass (bark and needles) compared with spruce biomass. The values of (−)-epicatechin, myricetin, quercetin, isorhamnetin, and apigenin quantified in spruce and fir biomass were lower compared with the data reported in the literature for spruce, fir, and pinus needle biomass [100,105].

Among the quantified phenolic acids, important amounts of 3,4-dihydroxybenzoic, 4-hydroxybenzoic, ferulic, syringic, and p-coumaric acids were quantified in spruce and fir biomass. Higher amounts of 3,4-dihydroxybenzoic, 4-hydroxybenzoic, chlorogenic, and p-coumaric acids correspond to needle biomass, while higher amounts of ferulic and syringic acids and CAPE correspond to bark biomass. The amounts of the analyzed individual phenolic acids were lower compared with those reported in the literature for spruce, fir, and pinus needle biomass [100,105]. Stilbene t-resveratrol was quantified in higher amounts in spruce biomass compared with fir biomass, with the obtained values being comparable with those reported in the literature for the *Pinus* sp. needles [105], but much lower than those reported for spruce needles [100] (see Table S4).

As previously mentioned, catechin, epicatechin, 4-hydroxybenzoic acid, rutin, myricetin, p-cumaric acid, kaempherol, and apigenin were the main polyphenols found in the selected samples (in quantities around 100 µg/g). These substances can impart the following bioactive properties to the extracts: antioxidant, cytoprotective, anti-inflammatory, anticarcinogenic, antidiabetic, antiasthmatic, antiallergic, hypocholesterolemic, neuroprotective, or cardioprotective activity. Catechins possess significant antioxidant effects and strong activity against several pathogens, including bacteria, viruses, parasites, and fungi [106]. Modulation of the metabolism of nitric oxide and reactive nitrogen species, which results in the preservation and improvement of the endothelial function of arterial vessels, was demonstrated for epicatechin [107]. Rutin display neuroprotective activity on brain ischemia, sedative and anticonvulsant effects, and suppress the activity of pro-inflammatory cytokines by decreasing the production of TNF-α and IL-1β in microglia, an effect that seems to be useful in the treatment of Alzheimer’s disease, by preventing the cytotoxicity of β-amyloid oligomers [108].

Ferulic acid demonstrated antioxidant, anti-inflammatory, antiviral, antiallergic, antimicrobial, antithrombotic, anticarcinogenic, and hepatoprotective activity; vasodilatory activity; modulation of the activity of some enzymes; and the activation of transcriptional and gene expression factors [109]. It can prevent oxidative damage and amyloid pathology in Alzheimer’s disease by degrading the amyloid structures [110]. The displayed antidiabetic effect may be related to the reduction in oxidative stress, which could help the β-cells of the pancreas to secrete more insulin [109].

Myricetin displayed anti-proliferative activity against acute leukemia HL-60 cells [111] as well as protective activity against skin cancer by strongly inhibiting tumor and neoplastic cell transformation induced by the promoter by restricting the activity of MEK kinases, JAK1, AKT, and MKK4 [112]. The hypotensive action and the potential to modulate the immune system was also demonstrated by in vivo studies [113]. Moreover, myricetin showed a strong analgesic effect by reducing the ionas current in calcium channels [114] and presents several benefits related to the central nervous system, including protection against Parkinson’s and Alzheimer’s diseases [111].

Besides showing a significant antioxidant effect, 4-hydroxybenzoic acid exhibits antimicrobial activity against Gram-positive and Gram-negative bacteria [115]. P-coumaric acid has a higher bioavailability compared to other phenolic acids and can be absorbed in all sectors of the gastrointestinal tract. p-Coumaric acid shows antidiabetic activity by reducing the intestinal absorption of dietary carbohydrates and also has a strong inhibitory effect on the enzymes involved in glucose metabolism (amylase and glucosidase) while stimulating insulin secretion [116].

The reported biological properties of apigenin include anti-oxidant, anti-mutagenic, anti-carcinogenic, anti-inflammatory, anti-proliferative, and anti-progressive activity. Apigenyn is an effective anti-inflammatory agent and the strongest inhibitor of transcriptional activation regarding both inducible cyclooxygenase (COX-2) and inducible nitric oxide synthase (iNOS) in cells activated with lipopolysaccharide (LPS), reducing nitric oxide production and inhibiting the production of pro-inflammatory cytokines in lipopolysaccharide-stimulated human monocytes [117]. Also, apigenyn is reportedly able to inhibit several viruses, including enterovirus 71 (EV71), herpes simplex virus HSV-1 and HSV-2, hepatitis C virus, and influenza virus [118].

Pearson correlation analysis further elucidated the bioactive characteristics (TP, TF, and AA), sum of the individual phenolic acids (Σ Phenolic acids), and sum of the individual flavonoids (Σ Flavonoids) (Figure S1), showing positive and negative correlations. Strong positive correlations (coefficients higher than 0.5) were observed between the bioactive characteristics and Σ Flavonoids, demonstrating the major contribution of the flavonoids to the general bioactive characteristics (TP, TF, and AA) of the studied coniferous biomass.

### 3.3. Non-Target HRMS Analysis for the Identification of Additional Bioactive Compounds in Coniferous Barks and Needles Extracts

A non-target HRMS analysis was performed in order to identify other bioactive compounds and specialized metabolites that occur in coniferous bark and needles, which are likely responsible for their bioactive properties. Data processing analysis using Compound Discoverer software with an untargeted metabolomics working template was performed, which involves a defined untargeted workflow that includes RT alignment, molecular formula prediction, the evaluation of adducts, the assignment and comparison of fragmentation pattern, background annotation, and automated library and database searches for identification purposes (mzCloud (ddMS2), Chemspider, MzVault, and Mass List Matches) [119]. Based on Compound Discoverer processing results including formula, molecular mass, accurate mass, retention time, *m*/*z* adducts, their areas, and intensities, and also using the ChemSpider and PubChem online databases and data available in the literature, we identified 86 compounds in the spruce and bark and needles extracts. In particular, six main classes of phytochemical compounds, such as phenolic acids, flavonoids, stilbenes, terpenes, lignans, carboxylic acids, and other bioactive compounds (secoiridoids, indanes), were identified in coniferous bark and needle extracts. The name, molecular formula, retention time, exact mass and accurate mass of *m*/*z* adduct ions, and MS/MS fragment ions in negative ESI mode of the main identified bioactive phytochemical compounds identified in spruce (*P. abies* L., H. Karst.) and fir (*A. alba* Mill.) biomass (bark and needles) by non-target HRMS analysis combined with Compound Discoverer data analysis are shown in Table 2, while the pharmacological activities of the bioactive compounds are presented in Table S5.

The most frequently identified bioactive compounds were flavonoids, which play important roles for human health due to their pharmacological activities, including antioxidant, antidiabetic, anti-inflammatory, antibacterial, anti-carcinogenic, neuroprotective, and estrogenic effects [120,121,122,123,124]. Thus, numerous flavonoids have been identified in spruce and fir biomass (bark and needles), including the following: flavanols (epigallocatechin, dihydrokaempferol, cedeodarin, taxifolin, and derivatives), flavones (tricin, vitexin, vitexin-2-o-rhamnoside, myricetin-3-o-rhamnoside, kaempferol-3-rutinoside, kaempferol/luteolin-o-glucoside), isoflavones (afromosin, pratensein, glycitein, daidzein), flavanones (liquiritigenin, pinostrobin), chalcones (phloretin and phloretin-2-glucoside), and proanthocyanidins (proanthocyanidin, procyanidin B1/B2, procyanidin A2). Other bioactive compounds identified in the coniferous biomass included phenol phytochemicals, such as phenolic acids (quinic, shikimic, salicylic, vanillic, caffeic, synaptic, homovanilic) and derivatives (caffeoylquinic and 3-p-coumaroylquinic, caffeoylshikimic, 4-methoxycinnamic and toluic acids, and ferulic and vanillic acid glucosides), with antioxidant, antimicrobial, anti-inflammatory, neuroprotective, anticarcinogenic, and antidiabetic properties [125,126,127,128,129,130]. Astringin, piceatannol glucoside, t-piceid, combretastatin A-4, t-isorhapontin, t-isorhapontigenin, and piceatannol represent the main stilbenes identified in the coniferous biomass, which exert various biological activities, including cardioprotective, neuroprotective, anti-inflammatory, antitumor, anti-diabetic [131], and antimicrobial effects [132] and also the inhibition of lipid peroxidation and scavenging of peroxyl radicals [133]. In the studied extracts, we also identified terpenic compounds (e.g., carnosic acid, carnosol, rosmanol derivatives, toosendanin, vernodalin), lignans and lignan derivatives (pinoresinol, sesquipinsapol B, lariciıesinol), carboxilic acids and derivatives (suberic, sebacic, azelaic, and retinoic acids), and other classes of bioactive compounds, such as glucosides, secoiridoids, indanes, and iridoids with antioxidative, anti-inflammatory, analgesic, and anticancer properties (See Table S5 for detailed biological activities).

**Figure 2:**
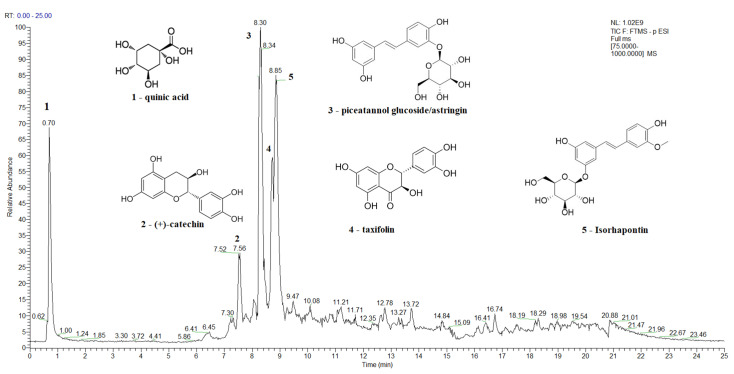
A total ion current (TIC) chromatogram showing the identification of the main bioactive compounds in spruce bark extract using UHPLC–MS/MS detection in negative ionization mode.

A Total Ion Current (TIC) chromatogram of the spruce bark extract in the negative ion mode, covering a scan range between 75 and 1000 *m*/*z*, is shown in Figure 2, while the TIC chromatograms of the main bioactive compounds identified in spruce needles, fir bark, and fir needles extracts are presented in Figure S2, Figure S3, and Figure S4, respectively. The major identified compounds in spruce bark extracts (Figure 2) were quinic acid, (+)-catechin, astringin, taxifolin, and isorhapontin, while quinic, shikimic, and toluic acids and (+)-catechin were the major identified compounds in the spruce needle extracts (Figure S2). In the case of the studied fir bark and needles, quinic and shikimic acids, (+)-catechin, 7-deoxyloganic acid glucopyranosyl ester, lucidumoside A, diosbulbinoside F, combrestatatin A5, pinoresinol, carnosol, azelaic, and etienic acids were identified in the fir bark extracts, while quinic acid, epigallocatechin, (+)-catechin, syringin, sacranoside A, and denipride were identified in the fir needle extracts.

Quinic and shikimic acids show antioxidant, antimicrobial, anti-inflammatory, and neuroprotective effects; hence, they are used as industrial precursors for the synthesis of oseltamivir phosphate, known as the antiinfluenza drug Tamiflu, which is used to treat influenza A and B [125,126]. Taxifolin, azelaic, and etienic acids exhibit strong antioxidant, anti-inflammatory, antiviral, antitumor, antibacterial, and enzyme-inhibiting activities [124,134]; lucidumoside A was shown to have antioxidant activity against the hemolysis of red blood cells induced by free radicals and antiviral activity [135]; carnosol; and antioxidative, anti-inflammatory, and antiproliferative activity, while carnosol and carnosic acid shows antioxidant and antiproliferative activities [136], and pinoresinol is a plant lignan with anticancer, hypoglycemic, and antifungal activities [137]. Diosbulbinoside F is a compound identified in the rhizome of *D. bulbifera* that has been used in folk and traditional Chinese medicine for the treatment of thyroid diseases and cancer [138]. Syringin has significant anti-inflammatory activity [139], epigallocatechin is an effective radical scavenger that protects against nerve cell damage [140], sacranoside A has anti-HCV activity and represents a potential drug candidate for antiviral activity against HCV “https://pubchem.ncbi.nlm.nih.gov/ (accessed on 7 June 2023)” , and denipride has a positive impact on the central nervous system and has been studied for its potential use in treating neurological disorders such as Parkinson’s disease “https://pubchem.ncbi.nlm.nih.gov/ (accessed on 7 June 2023)”.

### 3.4. Multivariate Statistical Analysis for the Identification of the Specific Biomarkers of Picea abies L., H. Karst., and Abies alba Mill. Bark and Needles Extracts

**Figure 3:**
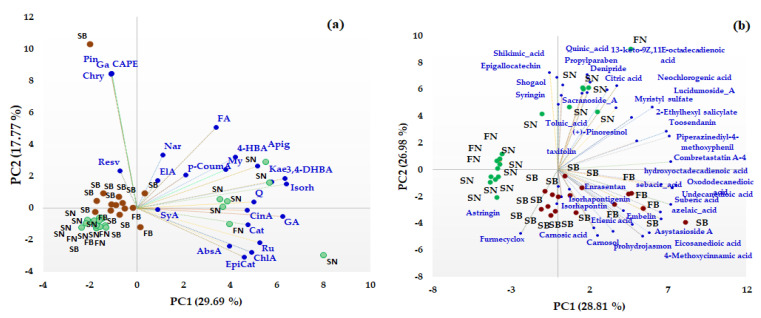
PCA results (scores and loading biplots) of different coniferous extracts (spruce bark—SB, spruce needles—SN, fir bark—FB, and fir needles—FN) based on quantified phenolic compound biomarkers (**a**) and non-targeted HRMS screening analysis (**b**).

Unsupervised multivariate methods, including Principal Component Analysis (PCA) and heat map analysis (HMA), were used in order to differentiate between different coniferous biomass (barks and needles of *Picea abies* L., H. Karst., and *Abies alba* Mill.) based on both the target and non-target HRMS profiling of the bioactive compounds. First, PCA was performed to facilitate an exploratory analysis of the data related to the quantitative data of some phenolic acids, flavonoids, and t-resveratrol (data presented in Table 1) and also for semiquantitative data (the area corresponding to the main representative signals in the HRMS spectra) obtained from non-target HRMS analysis (data presented in Table 2) in order to identify specific biomarkers for each type of coniferous extract. The distribution of the studied extracts in the PC1-PC2 scores plot is presented in Figure 3. The first two components of the PCA model accounted for 47.46% of the variance pertaining to the targeted analysis (Figure 3a) and 55.79% of the variance for the non-targeted screening analysis (Figure 3b), with a higher contribution being offered by PC1 compared to PC2 (in both cases).

From the PCA analysis based on both quantitative and not-targeted screening HRMS data, a clear discrimination between the bark and needles coniferous biomass can be observed. Based on quantitative data (Figure 3a), the majority of the quantified phenolic compounds (gallic (GA), cinnamic (CinA), chlorogenic (ChlA), 3,4-dihydroxybenzoic (DHyB), 4-hydroxybenzoic (HyB and abscisic (AbsA) acids, apigenin (Apig), kaempferol (Kae), isorhamnetin (Isorh), quercetin (Q), rutin (Ru), (+)-catechin (Cat), and (−)-epicatechin (EpiCat)) were distributed towards the needle extracts that contain higher amounts of the compounds, which are grouped on the right side of the PC1-PC2 score graph. *t*-Resveratrol (Resv), pinocembrin (Pin), caffeic acid phenethyl ester (CAPE), galangin (Ga), chrysin (Chry), naringin (Nar), ferulic (FA), and Ellagic (ElA) acids represent specific biomarkers of the bark extracts. Also, based on non-targeted screening HRMS data, the scores plot indicates a clear discrimination between the needle and bark extracts of *Picea abies* L., H. Karst., and *Abies alba* Mill. Thus, phytochemical bioactive compounds, such as astringin, isorhapontin, isorhapontigenin, carnosol, carnosic acid, combretastatin A, asistoside A, enrasentan, embelin, prohydrojasmon and sebacid, suberic, azelaic, 4-metoxycinnamic, eicosanedioic, and undecanedioic acids, represent specific phytochemical biomarkers of bark, while syringin, epigallocatechin, taxifolin, pinoresinol, shogaol, sacranoside A, lucidumoside A, denipride, toosendanin, myristil sulphate, diosbulbinoside F and quinic, shikimic, neochlorogenic, toluic, citric, and 13-keto-9Z,11E-octadecadienoic acids are phytochemical biomarkers of needles. No discrimination was observed between the bark of *Picea abies* L., H. Karst., and *Abies alba* Mill. or between the needles of *Picea abies* L., H. Karst., and *Abies alba* Mill., even if some bioactive phytochemical compounds were identified only in spruce or fir biomass (see Table S5).

**Figure 4:**
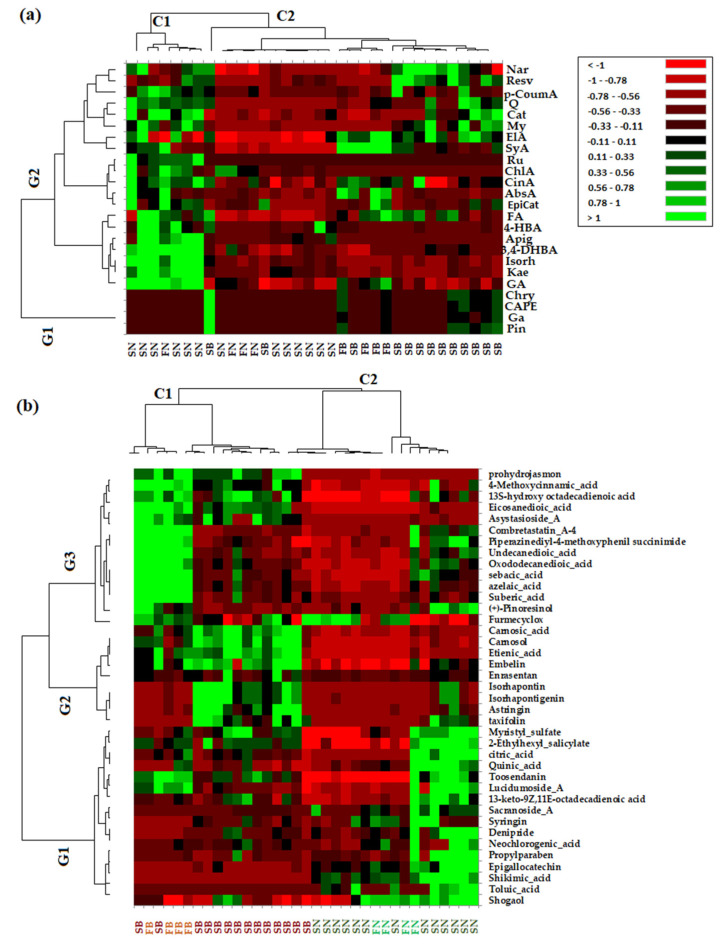
A heat map of discriminant features according to the different types of coniferous biomass (spruce bark—SB, spruce needles—SN, fir bark—FB, and fir needles—FN) based on quantified phenolic compounds biomarkers (**a**) and non-targeted HRMS screening analysis (**b**). Red and green cells correspond to low and high compound levels, respectively.

In order to confirm the PCA analysis and to extract as much information as possible from the acquired data, both the quantitative data of phenolic compounds and non-targeted data (which refers to the greater number of the phytochemical bioactive compounds identified in the needles and bark extracts of *Picea abies* L., H. Karst., and *Abies alba* Mill.) were used to obtain the heat map profiles (Figure 4).

From the HMA, it can be observed that the obtained results are in agreement with those obtained via PCA. Thus, as can be seen in Figure 4a, based on targeted data, the investigated coniferous extracts were clustered into two main clusters corresponding to needles (C1) and bark (C2) biomass, while the quantified variables were grouped into two main groups, with the first (G1) corresponding to the bark phytochemical compounds and the second (G2) being composed by the main tree subclusters that grouped phytochemical compounds representative of both needles and bark biomass. The heat map profiles based on the non-targeted HRMS data of the investigated coniferous extracts (Figure 4b) indicate two main clusters corresponding to bark (C1) and needles (C2) biomass, respectively, while also tentatively indicating subclusters for bark biomass corresponding to *Picea abies* L., H. Karst., and *Abies alba* Mill. (Figure 4b). The non-targeted identified variables were grouped into three main clusters corresponding to specific needle phytochemical biomarkers (G1), bark phytochemical biomarkers (G2), and both bark and needle phytochemical biomarkers (G3).

### 3.5. Antimicrobial Activity

The infectious process at the level of the skin involves, in the first stage, predominantly Gram-positive bacteria, such as *S. aureus*, *S. epidermidis*, *E. faecalis*, etc. Subsequently, Gram-negative bacteria such as *P. aeruginosa*, *A. baumannii/haemolyticus*, *E. coli*, *P. mirabilis* become involved when a chronic wound develops [141]. Yeast strains, e.g., species of *Candida* sp., can also be detected in infected wounds [142].

**Figure 5:**
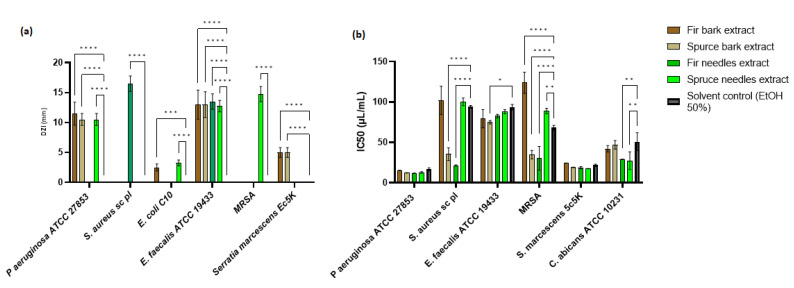
Antimicrobial activity of bark and needles extracts: (**a**) Mean of DIZ exhibited by hydroalcoholic extracts of *P. abies* and *A. alba* (needles and barks) vs. solvent control (ethanol 50%) and (**b**) IC50 related to microbial cell viability in the presence of *P. abies* and *A. alba* extracts (* *p* < 0.05, ** *p* < 0.01, *** *p* < 0.001, **** *p* < 0.0001).

Among the four extracts studied, *P. abies* needles extract showed a significant inhibition zone for *P. aeruginosa* (*p* < 0.0001), *E. coli* (*p* < 0.0001), *E. faecalis* (*p* < 0.0001), and MRSA (*p* < 0.0.0001) strains, while *A. alba* bark extract showed a significant inhibition zone against *P. aeruginosa* (*p* < 0.0.0001), *E. coli* (*p* < 0.0.001), *E. faecalis* (*p* < 0.0.0001), and *S. marcescens* (*p* < 0.0.0001) (Figure 5a). The antimicrobial activity of the *A. alba* needles extract was evident only against *S. aureus* sc and *E. faecalis* strains, while *P. abies* bark extract showed activity against *P. aeruginosa, E. faecalis*, and *S. marcescens* strains. Regarding the diameters of inhibition zones (DIZs), the antibacterial activities of extracts ranged between 2.5 and 16.5 mm. Maximum DIZ was observed for the *A. alba* needles extract against *S. aureus* sc (16.5 ± 1.29 mm), and minimum zone of inhibition was given by the *A. alba* bark extract against a clinical strain of *E. coli* (2.5 ± 0.58 mm). Statistical analysis was performed by comparing the antimicrobial activities between the extracts and the solvent control. The antimicrobial effect of the *P. abies* needles extract was better than the *A. alba* needles extract, probably due to phenolic content, while the extract from the *P. abies* bark was more active than the *A. alba* bark extract. The variation of the chemical components significantly changes the spectrum of antimicrobial activity.

The minimum inhibitory concentration (MIC) was used to accurately determine the plant extracts’ effectiveness. IC50 is a more precisely defined measure than MIC, and its standard error is readily calculable [143]. The concentration of plant extracts that inhibit the growth of half of a microbial inoculum (IC50) was estimated by calculating the percentage of viable cells with varying concentrations of plant extract or solvent control and fitting a probit inhibition curve to the results (Figure 5b).

The MIC values obtained from plant extracts exhibited antibacterial activity ranging between 15.625 and 250 µL/mL (Table S6). The highest antimicrobial activity was observed for the *A. alba* needles extract, followed by the *P. abies* needles extract. The *P. abies* needles extract was more active on Gram-negative bacteria strains than bark, but the last one showed broad-spectrum activity. The bark extracts had similar MICs. The *A. alba* needles gave a MIC value at 15.625 µL/mL against *P. aeruginosa*. In Table S6, the comparative antimicrobial activity between extracts and similar solvent concentrations is highlighted. The solvent had an antimicrobial activity between 62.5 and 250 µL/mL. *P. aeruginosa* and MRSA strains proved sensitive to all extracts compared to the solvent control. The *S. aureus* sc strain was sensitive to *P. abies* and *A. alba* needles extracts and *P. abies* bark extract. In the case of *E. faecalis*, it was observed that the antimicrobial activity was actually given by the solvent used, while the *S. marcescens* strain was only sensitive to *P. abies* needles extract. In the case of the *C. albicans* strain, for which no antimicrobial activity was observed following qualitative analysis, only the extracts from the needles showed activity.

The antimicrobial activity of these extracts may be attributable to a synergistic effect between all phenolic components [144]. In the case of the spruce bark extract, it was evident that the compounds like catechin, taxifolin, astringin, quinic acid, shikimic acid, and isorhapontin [145,146] have activity against *E. faecalis*, *S. thermophilus*, *Prevotella intermedia, Fusobacterium nucleatum*, *Streptococcus* spp. These compounds are also found in the other three extracts but in a smaller proportion. Probably due to the presence of compounds like procyanidine [147], sinapic acid [148], apigenin [149], lariciresinol [150], azelaic acid [151], and sinapic acid [129], the extracts of *A. alba* (needles and barks) demonstrated significantly increased antimicrobial activity compared to the extracts obtained from *P. abies.* Another main compound identified in spruce bark extract, isorhamnetin, is known for enhancing survival and reducing proinflammatory cytokine levels in the serum and lung tissue of *E. coli-*infected mice [152].

To facilitate a statistical analysis of the comparative antimicrobial activity between the extracts and the solvent control, the IC50 was evaluated. From Figure 5b, it can be seen that, in the case of *P. aeruginosa* and *S. marcescens*, the antimicrobial activity was not significantly different from that of the solvent (*p* > 0.05). Significant differences in antimicrobial activity were observed for *P. abies* bark and *A. alba* needles extracts (*p* < 0.0001) on *S. aureus* sc and MRSA strains. In the case of the *E. faecalis* strain, the *P. abies* bark extract demonstrated significant activity compared to the solvent used (*p* < 0.05), while the IC50 for *C. albicans* was significantly reduced by the extracts of needles (*p* < 0.01).

The association of microbial biofilms with the chronicity of infected wounds is an important topic. Microbial biofilms represent a physical barrier to wound healing, maintaining a constant inflammatory state, causing more lesions, and delaying tissue epithelization. Moreover, chronic inflammatory skin conditions are more susceptible to the development of pathogenic biofilms [153,154].

Infections associated with microbial biofilms are difficult to resolve [155]. The topical route allows the application of high concentrations of antibacterial agents directly to the site of infection, with low incidence of systemic side effects. The rise of antibiotic resistance and the emergence of multidrug-resistant (MDR) microorganisms are serious causes for concern, and the development of new tissue-biocompatible antimicrobial solutions has been slow [153]. Therefore, for these reasons, the anti-biofilm effect was studied by evaluating the reduction in microbial adhesion to the inert substrate.

From Table S6, it can be seen that the antibiofilm effect was manifested only for *S. aureus* sc, MRSA, and *C. albicans* strains. The antibiofilm activity against the *S. aureus* sc strain was given by the extracts obtained from needles and fir bark extract, while against MRSA, only *A. alba* bark extract showed activity. In the case of *C. albicans*, all extracts prevented microbial adhesion, with the most active being *A. alba* bark extract. It is likely that the fir bark extract contains other non-phenolic compounds that act on the formation of adhesins.

The extracts obtained from the barks had a similar anti-biofilm activity, but the extract from the fir needles was the most active.

### 3.6. Biocompatibility

**Figure 6:**
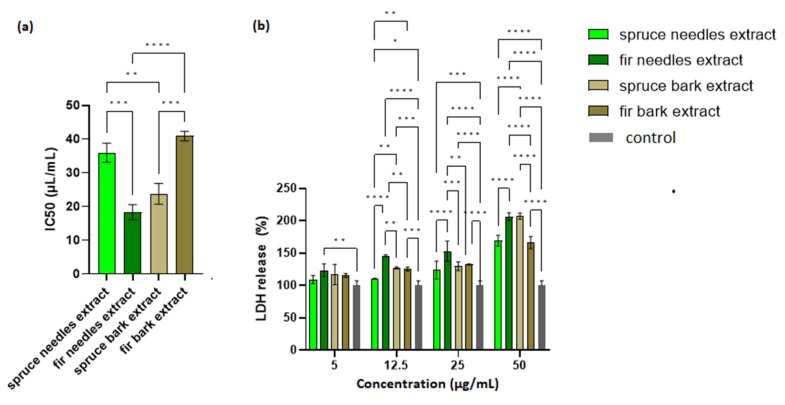
Biocompatibility of the extracts: (**a**) IC50 (µL/mL) and (**b**) LDH release (%) of HaCaT cells exposed to fir and spruce bark and needle extracts; significant differences (*p* < 0.05) in all comparisons are shown (* *p* < 0.05, ** *p* < 0.01, *** *p* < 0.001, **** *p* < 0.0001).

The possible toxic effects of the extracts were evaluated via MTT assay and LDH leakage assay on HaCaT cells. A cell viability assay conducted after 24 h of incubation showed that cell viability was the lowest for *A. alba* needles extract, followed by *P. abies* bark extract. As shown in Figure 6a, the IC50 values obtained for spruce needles and fir bark extracts did not differ significantly.

LDH is an intracellular enzyme that is released from cells where membrane integrity has been affected. Therefore, the measurement of LDH leakage in cell culture is the best indicator for the in vitro cytotoxicity [156]. It can be seen in Figure 6b that the extract with the lowest IC50 value (the fir needles extract) exhibited the largest amount of LDH released. Moreover, an increase in LDH release from cells in a manner dependent on the extract concentration was also observed. Thus, an optimization of the extract concentration in the final formulation based on a compromise between the cell viability and therapeutic properties is required.

Lower LDH leakage (for 25 µL/mL extract, Figure 6b) values were obtained with increasing values for antioxidant activity (DPPH assay). Thus, the antioxidant compounds could protect the integrity of eukaryotic cells (Pearson correlation, R^2^ = 0.9474, *p* < 0.05).

A relationship between cytotoxicity and antimicrobial activity was established through the Selectivity Index (SI) (Table S7). The SI values were calculated by the ratio of the IC50 and the MIC values for each microorganism (SI = IC50/MIC). Values over 1 represent elevated selectivity against microorganisms, whereas values below 1 indicate elevated toxicity towards HaCaT cells [157].

The extract from the spruce bark has an SI < 1 for all strains, both against MIC and the minimal biofilm eradication concentration (MBEC), which highlights that this extract was more toxic to eukaryotic cells than to microbial ones. The other extracts proved to be more cytotoxic against the *P. aeruginosa* strain compared to the eukaryotic ones, which suggests that the MIC and MBEC values can be selective against *P. aeruginosa* infections without affecting the HaCaT cells. This effect was also observed for the extracts from the needles in relation to the microbial adhesion of *S. aureus* sc pl cells. The selective antibiofilm effect was evident for the bark extract (MRSA and *C. albicans*) and spruce needles extract (*S. marcescens* and *C. albicans*). Therefore, we can conclude that the extracts could be used for the antimicrobial effect only in the case of monospecific *P. aeruginosa* infection, but spruce needles extract shows a broad-spectrum antibiofilm effect (*P. aeruginosa*, *S. aureus* sc pl, *S. marcescens* and *C. albicans*).

### 3.7. Anti-Hemolytic Activity

**Figure 7:**
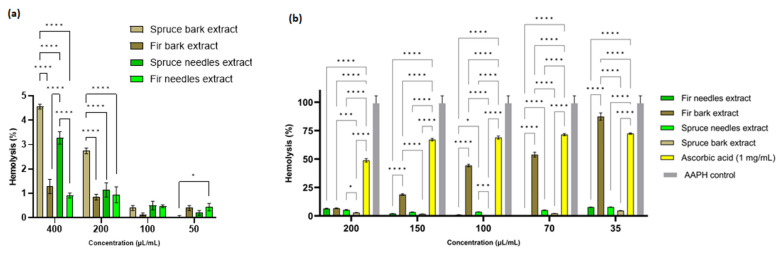
Anti-hemolytic activity of bark and needles extracts: (**a**) Hemolysis (%) induced by *Picea abies* and *Abies alba* extracts and (**b**) Antihemolytic activity of fir and spruce extracts against the AAPH-induced oxidative hemolysis of ram erythrocytes (* *p* < 0.05, *** *p* < 0.001, **** *p* < 0.0001).

In order to select the extract concentrations for the anti-hemolytic activity evaluation, the hemolysis index was determined and the extract concentrations below 200 µL/mL were selected. From Figure 7a, it is evident that at the concentration of 400 µL/mL, the spruce bark and needles extracts are slightly hemolytic, but with values below 5%. The differences are significant between the bark of *P. abies* and *A. alba* extracts (*p* < 0.0001), *A. alba* being non-hemolytic at all concentrations used. Therefore, extracts from *A. alba* could be used as active principles in open wounds while *P. abies* becomes hemocompatible at concentrations lower than 200 µL/mL. By comparison with the antimicrobial activity, it seems that the MIC and MBEC values are lower than the concentrations that induce hemolysis. Thus, these extracts can be used as active antimicrobial principles for infected wounds. Catechin possesses an accentuated hemolytic effect according to Veiko et al. [158], so due to the high content of catechin in spruce bark extract, the high hemolytic index compared to the other extracts could be explained.

The antihemolytic activity of the fir and spruce extracts was evaluated by measuring the degree of hemolysis induced in ram erythrocytes by free radicals generated by AAPH. The pretreatment of RBCs with different doses (35–200 µL/mL) of the two needle extracts significantly attenuated the concentration of AAPH-induced hemolysis (Figure 7b). The results clearly show that all four extracts were more effective than ascorbic acid in reducing AAPH-induced hemolysis. Indeed, the hemolysis inhibition percentages for the *P. abies* bark extract were almost 100% inhibition for all concentrations used, while the different concentrations of ascorbic acid (1 mg/mL was used as a standard) gave an inhibition percentage of 51.17 ± 1.53% to 27.61 ± 0.58% (Figure 7b). The inhibition of hemolysis by the extracts indicates that these extracts probably enhanced the antioxidant capacity of erythrocytes to quench free radicals and thereby attenuate oxidative hemolysis [94].

Basically, it was observed that when the erythrocytes were treated with AAPH and the four extracts, a significant reduction (*p* < 0.0001) in hemolysis was observed compared to the cells treated only with phosphate-buffered saline (value set as 100% hemolysis) (*p* < 0.0001).

The antihemolytic activity was probably due to various phenolic compounds in the extracts, known as phenolic hydrogen atom donors. They help to stabilize free radicals through their antioxidant activity, increasing the resistance of erythrocytes to oxidative stress. Moreover, the phenolic components in the composition of the extracts could donate one or more electrons to neutralize the AAPH radical, leading to the inhibition of hemolysis. According to Balderrama-Carmona et al. [159], polyphenols show the ability to interact with the hydrophilic section of the lipid membrane, causing changes in the packing arrangement of the lipid polar ends.

The results of the antihemolytic effect were correlated with those obtained for the antioxidant activity achieved via the DPPH method. Thus, at a concentration of 35 µL/mL extract, it was observed that the greatest reduction in hemolysis was for the extract from spruce needles, followed by the extract from fir needles, spruce bark, and fir bark. Among these extracts, spruce needles had a higher content of flavonoids compared to the other extracts.

Flavonoids, as well as other polyphenols, protect erythrocyte cell membranes by interacting with membrane phospholipids, thus preventing lipid damage [160]. Flavonoids also promote Van der Waals interactions in the lipid bilayer and can be a source of membrane stabilization, protecting erythrocytes from damage generated by free radicals [161].

### 3.8. P–LD Runs against PI3Kγ

**Figure 8:**
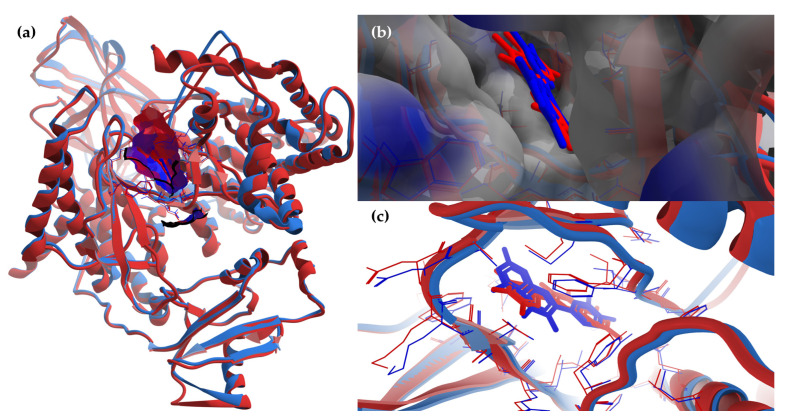
The superimposing of the structural files (3D) for the two RCSB PDB entries for PI3Kγ with the co-crystalized ligands (1E8W’s structural components are colored in blue—PI3Kγ and quercetin; 1E90’s structural components are colored in red—PI3Kγ and myricetin; macromolecules are depicted as wireframes, with their secondary structures being drawn as cartoons, while ligands are figured as sticks; to ensure a clear image, only the amino acid residues around a distance of 6 Å from ligands are shown). (**a**) General view of the molecular surface (80% transparency) of the ATP-binding pocket (with the two co-crystalized ligands inside), colored by hydrophobicity (residue atoms are colored according to the hydropathy index of amino acids). To ensure a clear image, the molecular surface is shown only for the structure chosen for docking: 1E8W. (**b**) Detailed view of the ATP-binding pocket with the two co-crystalized ligands inside, with molecular surface (80% transparency) colored according to the electrostatic partial charges of residues (blue corresponds to the positive charge, red corresponds to the negative charge). To ensure a clear image, the molecular surface is shown only for the structure chosen for docking: 1E8W. (**c**) Simplified detailed view of the two co-crystalized ligands and their targets (the region of the ATP binding pocket).

The superimposing of the structural files of the two RCSB PDB entries for PI3Kγ (1E8W with resolution = 2.50 Å and 1E90 with resolution = 2.70 Å) shows some folding differences, including in the region of the ATP binding pocket, where the co-crystalized ligands bind (Figure 8); therefore, choosing structures with a higher resolution is a critical requirement for the accuracy of docking results.

**Figure 9:**
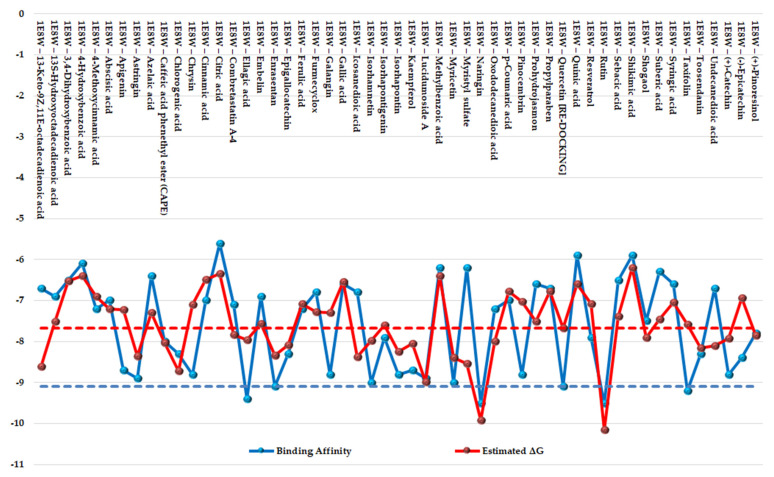
Comparative docking: graphical depiction of data for the best-ranked poses of the ligands (with blue showing the PyRx w/Autodock Vina results; red showing the SwissDock w/EADock DSS results). The vertical *y*-axis is negative. A lower value indicates a better binder. The blue color represents the values of BA (kcal/mol). The red color represents values of ΔG (kcal/mol). The values of BA and ΔG of quercetin (control/reference ligand) are depicted as horizontal dashed lines.

**Figure 10:**
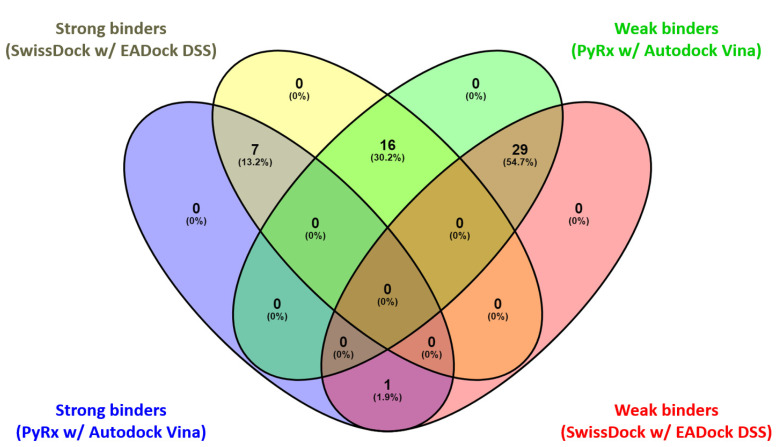
Venn diagram: comparative graphic depiction of the results generated by the two P–LD runs for the best-ranked poses of the ligands. Venny 2.1 “https://bioinfogp.cnb.csic.es/tools/venny/index.html (accessed on 25 May 2023)” was used to analyze the output of the P–LD runs (matchings are shown as numbers and percentage).

The results of the two comparative P–LD runs (against 53 compounds, quercetin—the reference compound—is included) are depicted briefly in Figure 9 and Figure 10 for the best poses for each docking run. The P–LD runs carried-out with PyRx—Python Prescription v.0.9.7 software using Autodock Vina as a docking algorithm (PyRx w/Autodock Vina) were used as ranking criteria for the best pose of each ligand; the zero values of the RMSD terms (RMSD/lb and RMSD/ub) combined with the lowest value of the Binding Affinity (BA in kcal/mol). In the case of the P–LD run carried-out with SwissDock web-service using the EADock DSS algorithm (SwissDock w/EADock DSS), the best-ranked poses were selected using the FullFitness ranking system (kcal/mol) and Estimated ΔG (kcal/mol) values for the best binding affinity to the target. The full dataset used for the two P–LD runs is presented in the Supplementary Materials (Supplementary Table S8).

Considering the fact that quercetin (present in the 1E8W entry) and myricetin (present in the 1E90 entry) are the co-crystalized ligands and known inhibitors of PI3Kγ [73], their values were considered as quasi-empirical thresholds to separate the strong binders from the weaker binders in the two P–LD runs. In the PyRx w/Autodock Vina P–LD run, quercetin (BA = −9.1 kcal/mol) slightly outperformed myricetin (BA = −9.0 kcal/mol), and the threshold was set to BA = −9.0 kcal/mol. In the SwissDock w/EADock DSS P–LD run, quercetin (ΔG = −7.68 kcal/mol) was found as a weaker inhibitor than myricetin (ΔG = −8.40 kcal/mol), and the threshold was set to ΔG = −7.68 kcal/mol. Therefore, for the PyRx w/Autodock Vina P–LD run, the best binder was found to be naringin (BA = −9.50 kcal/mol), followed in order by rutin, ellagic acid, quercetin (control/reference compound), isorhamnetin, taxifolin, enrasentan, and myricetin. The best binder found in the SwissDock w/EADock DSS P–LD run was rutin (ΔG = −10.16 kcal/mol), followed in order by naringin, lucidumoside A, chlorogenic acid, 13-keto-9Z,11e-octadecadienoic acid, myristyl sulfate, myricetin, icosanedioic acid, astringin, enrasentan, toosendanin, undecanedioic acid, epigallocatechin, kaempferol, caffeic acid phenethyl ester (CAPE), ellagic acid, oxododecanedioic acid, isorhamnetin, shogaol, (+)-pinoresinol, combretastatin A-4, and quercetin (control/reference compound). The major differences between the results generated by the two algorithms (Autodock Vina and EADock DSS) are shown in Figure 10.

As seen in Figure 9 and Figure 10, there are some differences between the results generated with the two docking algorithms, but the Venn diagram (Figure 10) output identified the following four categories of matchings (consistencies and discrepancies alike): (i) 1 common element (1.9%) in “Strong binders (PyRx w/Autodock Vina)” and “Weak binders (SwissDock w/EADock DSS)”: taxifolin (note: discrepancy between the results generated by the two algorithms); (ii) 7 common elements (13.2%) in “Strong binders (PyRx w/Autodock Vina)” and “ Strong binders (SwissDock w/EADock DSS)”: naringin, rutin, ellagic acid, quercetin, (control/reference compound), isorhamnetin, myricetin and enrasentan (note: consistency between the results generated by the two algorithms); (iii) 16 common elements (30.2%) in “Strong binders (SwissDock w/EADock DSS)” and “Weak binders (PyRx w/Autodock Vina)”: kaempferol, chlorogenic acid, caffeic acid phenethyl ester (CAPE), astringin, lucidumoside A, isorhapontin, epigallocatechin, toosendanin, (+)-pinoresinol, shogaol, oxododecanedioic acid, combretastatin A-4, icosanedioic acid, 13-keto-9Z,11e-octadecadienoic acid, undecanedioic acid, myristyl sulfate (note: discrepancy between the results generated by the two algorithms); (iv) 29 common elements (54.7%) in “Weak binders (PyRx w/Autodock Vina)” and “Weak binders (SwissDock w/EADock DSS)”: chrysin, galangin, pinocembrin, (+)-catechin, apigenin, (−)-epicatechin, resveratrol, ferulic acid, abscisic acid, cinnamic acid, p-coumaric acid, gallic acid, syringic acid, 3,4-dihydroxybenzoic acid, 4-hydroxybenzoic acid, isorhapontigenin, 4-methoxycinnamic acid, 13s-hydroxyoctadecadienoic acid, embelin, furmecyclox, propylparaben, prohydrojasmon, sebacic acid, azelaic acid, suberic acid, methylbenzoic acid, quinic acid, shikimic acid and citric acid (note: discrepancy between the results generated by the two algorithms).

**Figure 11:**
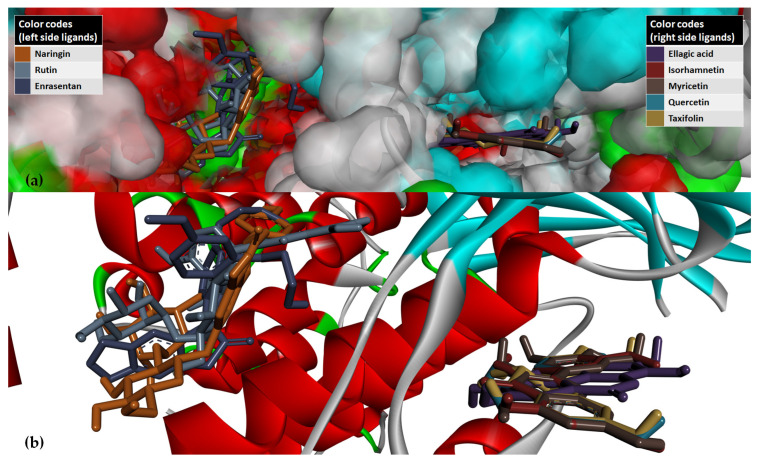
Graphical depiction of the eight best poses of the strongest binders based on results of the PyRx w/Autodock Vina P–LD run against PI3Kγ (PI3Kγ is depicted in the secondary structures drawn as cartoons, while ligands are depicted as sticks; to ensure a clear image, the distant amino acid residues have been hidden/cropped). (**a**) Detailed view of the ATP-binding pocket with docked ligands, with molecular surface (80% transparency) colored according to the parental color of the residues. (**b**) Simplified detailed view of binding modes of ligands in the region of the ATP binding pocket of PI3Kγ.

Overall, the two docking algorithms agreed regarding the docking results for the 36 analyzed ligands, representing 67.9% from all the investigated compounds (53 ligands). The seven strongest binders identified via consensus between the results of the PyRx w/Autodock Vina P–LD run and the SwissDock w/EADock DSS P–LD run and taxifolin (identified as strong binder only in the results of the PyRx w/Autodock Vina P–LD run, and also an analogs of quercetin) were analyzed further to determine their binding modes with PI3Kγ (Figure 11).

Figure 11 shows the two binding areas of ligands in the ATP binding pocket of PI3Kγ. The ligands with a greater molecular weight and a higher structural complexity (naringin, rutin, and enrasentan) bind in a different region than the ligands with a lower molecular weight (ellagic acid, quercetin, and analogs isorhamnetin, myricetin, and taxifolin), which are flat-type molecules. Moreover, from Figure 11a it can be observed that the docking cavity where the five flat-type molecules were fitted is tighter than the wider docking site of naringin, rutin, and enrasentan and therefore inaccessible to them.

Disregarding their binding patterns in the ATP binding pocket of PI3Kγ, the eight strong binders (naringin, rutin, ellagic acid, quercetin, isorhamnetin, myricetin, and enrasentan) may act as inhibitors of PI3Kγ. Therefore, those strong binders can be involved in the downregulation of the effectors of the PI3K/AKT signaling pathway and can exert anti-inflammatory, antioxidant, and autophagy regulatory effects during the healing process for the treatment the (chronic) wounds. The results of the P–LD runs against PI3Kγ correlate with the results regarding antioxidant activity obtained via the DPPH method and antihemolytic tests from a biomolecular mechanistic point of view. Moreover, as the flat-type inhibitors of PI3Kγ are typically non-selective [66], it is reasonable to assume that ellagic acid and quercetin and its analogs may also bind with PI3Kα (a class IA PI3K).

Phenolic compounds, such as those evaluated using the DPPH method, often exhibit antioxidant activity. Antioxidants can influence cellular signaling pathways, including the PI3K/AKT signaling pathway, by modulating ROS levels or directly affecting the activity of signaling molecules (PI3Kγ). Therefore, phenols with antioxidant properties may indirectly impact the PI3K/AKT signaling pathway through their effects on oxidative stress and redox signaling.

## 4. Conclusions

The major identified compounds in *Picea abies* L., H. Karst. biomass were (+)-catechin, astringin, taxifolin, isorhapontin, quinic, shikimic, and toluic acids, while (+)-catechin, epigallocatechin, quinic and shikimic acids, lucidumoside A, diosbulbinoside F, combrestatatin A5, sacranoside A, denipride, pinoresinol, carnosol, azelaic, and etienic acids were representative of *Abies alba* Mill. biomass. The studied extracts showed antimicrobial activity, especially for those isolated from infected wounds, with the *A. abies* needles extract being the most active. A major reason for the persistence of chronic wounds is the development of biofilms by the opportunistic pathogenic species. Thus, the prevention of the microbial biofilm’s formation and the antioxidant effect on ram erythrocytes and eukaryotic cell viability (IC50) encourages us to continue our research regarding the development of new products that use spruce needles extract as an active principle for the healing of the chronic wounds. The wet-lab assays were supported by the in silico technique of the molecular docking and revealed the mechanistic interactions of the selected phenolic compounds with PI3Kγ and their possible effects over the PI3K/AKT signaling pathway, as the PI3K/AKT signaling pathway and its interaction with ROS are linked to the healing process of (chronic) wounds [13,69]. Overall, the wet-lab assays combined with the in silico techniques intertwine in the study of bioactive natural compounds. The evaluation of antioxidant properties and the investigation of cellular signaling pathways like PI3K/AKT showcase the multifaceted nature of biochemistry and molecular biology research.

## Figures and Tables

**Table 1:** Phenolic compound content (µg/g, dry weight DW) of spruce (*P. abies* L., H. Karst.) and fir (*A. alba* Mill.) biomass (bark and needles).

Phenolic Compounds	Spruce Needles (*n* = 13)	Spruce Bark (*n* = 13)	Fir Needles (*n* = 4)	Fir Bark (*n* = 4)
**Phenolic acids and derivatives**				
Gallic acid	3.4–18.0/9.0 ± 5.2	2.2–7.1/5.1 ± 1.6	5.2–19.5/9.9 ± 6.5	7.4–11.6/9.2 ± 1.8
3,4-dihydroxybenzoic acid	2.4–112.7/42.4 ± 39.6	1.6–30.1/16.5 ± 8.6	10.2–83.6/36.2 ± 32.8	3.4–15.6/10.8 ± 5.9
4-hydroxybenzoic acid	3.1–300.0/68.2 ± 87.7	1.1–61.6/12.6 ± 16.7	3.4–49.6/16.8 ± 22.0	5.3–13.6/8.2 ± 3.9
Chlorogenic acid	0.7–53.9/8.0 ± 14.3	0.3–0.8/0.5 ± 0.1	4.9–25.1/11.9 ± 9.5	0.1–0.6/0.3 ± 0.2
Ferulic acid	2.6–153.4/32.6 ± 45.9	1.2–65.4/32.7 ± 20.6	2.5–45.3/15.4 ± 20.1	29.4–93.9/52.5 ± 28.5
Syringic acid	2.6–37.6/13.9 ± 12.6	7.9–82.2/28.2 ± 18.2	5.6–58.5/20.2 ± 25.6	78.7–91.3/86.6 ± 5.6
p-Coumaric acid	5.1–141.3/37.2 ± 40.1	0.6–373.0/55.3 ± 99.4	12.5–102.3/42.5 ± 41.9	2.0–31.8/16.1 ± 13.6
Ellagic acid	0.2–2.6/0.8 ± 0.8	0.4–2.9/1.5 ± 0.6	0.2–0.4/0.3 ± 0.1	1.5–3.3/2.5 ± 0.8
Abscisic acid	0.1–9.7/1.4 ± 2.6	0.1–3.1/0.7 ± 0.8	0.6–6.6/2.3 ± 2.9	0.7–7.9/4.0 ± 3.0
Cinnamic acid	4.4–34.7/10.8 ± 8.4	5.0–21.0/10.2 ± 4.6	5.9–15.0/9.7 ± 4.1	7.8–12.0/9.9 ± 1.8
Caffeic Acid Phenethyl Ester	0.2–1.9/0.5 ± 0.4	0.2–92.9/26.8 ± 33.8	0.2–0.5/0.4 ± 0.1	0.5–68.4/26.9 ± 32.8
∑ Phenolic acids	224.8	190.1	165.6	227.0
Flavonoids				
(+)-Catechin	108.7–1529.4/510.6 ± 478.3	48.5–1420.4/401.3 ± 424.8	94.0–894.5/332.5 ± 377.0	53.8–186.6/116.1 ± 60.6
(−)-Epicatechin	2.4–163.9/23.7 ± 43.5	2.6–39.5/14.1 ± 11.5	10.6–52.2/22.9 ± 19.7	8.2–78.9/33.5 ± 32.8
Myricetin	17.9–152.4/48.7 ± 42.3	17.3–144.8/50.8 ± 36.5	17.9–29.3/21.3 ± 5.4	17.5–19.8/18.3 ± 1.1
Naringin	0.1–1.0/0.4 ± 0.3	0.2–1.6/0.8 ± 0.5	0.00–0.3/0.1 ± 0.1	0.1–0.4/0.2 ± 0.1
Rutin	1.2–269.0/36.2 ± 72.6	0.1–2.0/0.8 ± 0.7	3.6–35.5/12.6 ± 15.3	NF
Quercitin	4.7–64.1/15.8 ± 16.4	5.3–55.1/15.8 ± 13.5	4.8–18.5/8.9 ± 6.5	4.8–15.3/10.9 ± 5.1
Kaempferol	11.1–142.2/55.6 ± 48.2	7.0–17.2/9.1 ± 3.5	10.5–53.9/22.4 ± 21.1	7.3–7.8/7.6 ± 0.2
Isorhamnetin	2.0–33.5/14.6 ± 12.8	0.7–6.3/1.9 ± 1.7	1.1–10.5/4.1 ± 4.3	0.6–1.0/0.8 ± 0.2
Apigenin	3.7–137.3/38.1 ± 42.6	0.4–10.6/2.4 ± 2.7	2.5–32.0/10.4 ± 14.5	0.6–1.2/0.8 ± 0.3
Pinocembrin	2.4–3.2/2.6 ± 0.3	2.5–69.3/9.9 ± 18.0	2.6–2.9/2.7 ± 0.2	2.6–8.9/5.1 ± 3.1
Chrysin	5.4–6.1/5.5 ± 0.2	5.4–54.2/10.6 ± 13.2	5.4–5.5/5.5 ± 0.02	5.5–9.0/6.9 ± 1.7
Galangin	13.8–14.3/14.0 ± 0.1	13.7–98.3/22.1 ± 23.0	13.9–14.0/14.0 ± 0.1	13.9–18.2/15.6 ± 2.1
∑ Flavonoids	766.0	539.6	457.4	215.5
Stilbenes				
t-Resveratrol	0.5–37.6/12.9 ± 10.4	0.2–146.6/41.7 ± 38.5	0.1–3.5/1.7 ± 1.5	0.5–2.5/1.5 ± 0.8

**Table 2:** The phytochemical bioactive compounds identified in spruce (*Picea abies* L., H. Karst.) and fir (*Abies alba* Mill.) biomass (bark and needles) via non-target HRMS analysis combined with Compound Discoverer data analysis.

No.	Compound	Retention Time [min]	Formula	Exact Mass	Accurate Mass [M-H]^−^	Mass Fragments
Phenolic acids and derivatives						
1′	Quinic acid	0.7	C_7_H_12_O_6_	192.0633	191.0552	85.0281, 93.0332
2′	Shikimic acid	0.84	C_7_H_10_O_5_	174.0528	173.0446	71.0124, 85.0281, 115.0024, 96.9587
3′	5-Caffeoylquinic acid (Neochlorogenic acid)	3.19	C_16_H_18_O_9_	354.095	353.0882	190.9864, 178.9482
4′	Salicylic acid	6.45	C_7_H_6_O_3_	138.0316	137.0231	108.0203, 136.0154, 79.5995
5′	Vanilic acid	6.73	C_8_H_8_O_4_	168.0422	167.0340	152.0105, 139.0025
6′	Ferulic acid glucoside	7.32	C_16_H_20_O_9_	356.1107	355.1037	307.1400, 193.0449
7′	3-p-coumaroylquinic acid/izomeri	7.53	C_16_H_18_O_8_	338.1002	337.0929	119.0532; 155.0404; 163.0424
8′	5-O-Caffeoylshikimic acid	7.55/8.52	C_16_H_16_O_8_	336.0844	335.0778	163.0391,135.0442
9′	Caffeic acid	8.00	C_9_H_8_O_4_	180.0422	179.0340	135.0439
10′	Acetyl salicylic acid	8.02	C_9_H_8_O_4_	180.0423	179.0341	119.9686,121.0282,122,0361
11′	Vanilic acid glucoside	8.18	C_14_H_18_O_9_	330.2890	329.0880	319.0461, 315.1236
12′	4-Methilbenzoic acid (toluic acid)—isomers	8.25	C_8_H_8_O_2_	136.0524	135.440	74.8001; 92.3021; 91.1004
13′	Sinapic acid	8.94	C_11_H_12_O_5_	224.0684	223.0609	79.7560, 95.9510, 118.9651
14′	4-Methoxycinnamic acid	8.96	C_10_H_10_O_3_	178.0629	177.0549	103.9189, 133.0283, 117.0334
15′	Coniferyl alcohol	9.07	C_10_H_12_O_3_	180.0787	179.0706	137.0232, 136.0157
16′	Homovanilic acid	9.31	C_9_H_10_O_4_	182.0579	181.0499	79.9559, 118.9650, 95.9509
Flavonoids and derivatives						
17′	Epigallocatechin	6.02	C_15_H_14_O_7_	305.0667	305.0668	191.0190
18′	Procyanidin B1/B2	7.33	C_30_H_26_O_12_	578.1424	577.1355	125.0231; 289.0719; 407.0774
19′	Taxifolin—3-O rhamnoside (isomers)	8.16/8.89	C_21_H_22_O_11_	450.1162	449.1091	151.0044, 285.0428,303.0514
20′	Phloretin	8.10/8.96/10.83	C_15_H_14_O_5_	274.0841	273.0768	93.0332, 121.0283
21′	Dihydrokaempferol (aromodendrin)	8.43	C_15_H_12_O_6_	288.063	287.0560	125.0231, 177.0548
22′	Taxifolin glucoside	8.71	C_21_H_22_O_12_	466.1111	465.1044	151.0027, 285.0406
23′	Phloretin—2-O glucoside	8.77	C_21_H_24_O_10_	436.1370	435.1297	273.0768; 179.0341; 125.0237
24′	Proanthocyanidin	8.81	C_31_H_28_O_12_	592.1581	591.1555	273.0768; 179.0341; 125.0237
25′	Procyanidin A2	9.25	C_30_H_24_O_12_	576.1267	575.1198	449.0, 423.4, 288.8591, 285.0405
26′	Tricin	8.73	C_17_H_14_O_7_	330.0740	329.0630	299.05634; 284.03290; 243.03044; 227.03470; 161.02370
27′	Taxifolin	8.75	C_15_H_12_O_7_	303.0510	303.0511	147.0440, 257.0814
28′	Vitexin-2-O-rhamnoside	8.98/9.66	C_27_H_30_O_14_	578.1636	577.1565	293.0826, 413.0883, 311.0551
29′	Cedeodarin	9.23	C_16_H_14_O_7_	318.0739	317.0666	165.0911, 71.0124, 89.0230
30′	Myricetin-3-O rhamnoside	9.46	C_21_H_20_O_12_	464.0955	463.0882	318.0314
31′	Kaempferol-3-O-rutinoside	9.93	C_27_H_30_O_15_	594.1585	593.1512	299.0559; 284.0328; 255.0299; 227.0346; 229.05032; 133.02834
32′	Kaempferol/luteolin-O-glucoside/isomers	9.95	C_21_H_20_O_11_	448.1006	447.0933	284.04077; 284.03299; 255.02995; 243.02979
33′	vitexin (apigenin 8-C-glucoside)/isovitexin	10.53	C_21_H_20_O_10_	432.1057	431.0984	341.0664; 269.0454; 240.0422; 197.0606
34′	Pratensein/Tectprogenin	11.90	C_16_H_12_O_6_	300.0634	299.0561	284.0331; 283.1001; 257.0461; 135.0075; 211.0399
35′	Afrormosin	11.51/15.21	C_17_H_14_O_5_	298.0842	297.0769	282.0535; 283.0569; 267.03021; 253.0507; 167.0499
36′	Liquiritigenin/liquiritigenin	12.54	C_15_H_12_O_4_	256.0736	255.06631	211.0764; 135.0077; 119.0490; 117.0333
37′	Pinostrobin	12.56	C_16_H_14_O_4_	270.0892	269.0819	179.0551
38′	Glycitein/biochanin A	12.87/13.92	C_16_H_12_O_5_	284.0684	283.0612	211.0383, 147.0143, 239.0326
39′	Daidzein	13.39	C_15_H_10_O_4_	254.0579	253.0505	208.0525, 223.0396, 133.0291, 132.0213, 180.575
40′	Rosmanol methyl ether	13.53	C_21_H_28_O_5_	360.1937	359.1864	351.2178; 269.1915
41′	3-Methyl galangin	13.92	C_16_H_12_O_5_	284.0685	283.0612	269.0458; 268.0379; 240.0430; 223.0399; 211.0403; 148.01559
Stilbenes and derivatives						
42′	Astringin	8.29	C_20_H_22_O_9_	406.1263	405.1189	243.0645, 241.0499,242.0522
43′	Piceatannol glucoside	8.69	C_20_H_22_O_9_	406.1264	405.1189	243.0659; 175.0780; 199.0754
44′	*t*-Piceid	8.75	C_20_H_22_O_8_	390.1314	389.1242	227.07088; 185.05998; 143.04909; 228.07426
45′	Combretastatin A-4 (isomers)	8.85	C_18_H_20_O_5_	316.1310	315.1236	301, 1415; 141.5800
46′	*t*-Isorhapontin,	8.85	C_21_H_24_O_9_	420.14203	419.1347	258.08515; 257.08182; 241.05034; 204.04207
47′	*t*-Isorhapontigenin	8.86	C_15_H_14_O_4_	258.08921	257.0819	241.05038; 125.02316; 175.03926; 217.05028; 175.03926
48′	Piceatannol	8.92	C_14_H_12_O_4_	244.07356	243.0660	177.0548, 173.0811, 137.0233
Terpenes						
49′	Toosendanin (isomers)	9.04	C_30_H_38_O_11_	574.2419	573.2341	165.0549, 195.0657
50′	Vernodalin	9.36	C_19_H_20_O_7_	360.1209	359.1137	119.0498, 145.0284, 165.0911
51′	Inulicin (isomers)	10.08/11.07/16.07	C_17_H_24_O_5_	308.1623	307.1551	71.0124, 146.9501, 174.9551
52′	Podocarpic acid	12.62	C_17_H_22_O_3_	274.1569	273.14964	273.1496
53′	Carnosol (isomers)	12.76/13.18	C_20_H_26_O_4_	330.1831	329.1758	329.1738, 227.1436
54′	Carnosic acid (isomers)	11.60/13.10/13.73	C_20_H_28_O_4_	332.1987	331.1915	331.1915, 112.9843, 146.9601
55′	Rosmaridiphenol/izomer	14.84/16.76	C_20_H_28_O_3_	316.2039	315.1966	315.1967; 297.1859; 235.1487
Lignans and derivatives						
56′	Hydroxymatairesinol (isomers)	8.89/9.25/9.51	C_20_H_22_O_7_	374.13655	373.1293	152.0343, 137.0233
57′	(+)-Pinoresinol	10.06	C_20_H_22_O_6_	358.1416	357.1343	165.0912
58′	Sesquipinsapol B	10.10	C30H36O9	540.2360	539.2286	509.2183; 391.1662; 195.0657
59′	Lariciıesinol/isolariciresinol	11.52/11.71	C_20_H_24_O_6_	360.15729	359.1477	310.9311, 139.0025, 240.9285
Carboxilic acids and derivatives						
60′	Citric acid	1.06	C_6_H_8_O_7_	192.0270	191.0189	111.0075, 87.0074, 85.0281
61′	Suberic acid	8.91	C_8_H_14_O_4_	174.0892	173.0810	83.0124,109.2006, 111.1001
62′	Azelaic acid	9.76	C_9_H_16_O_4_	188.1048	187.0975	97.0281; 123.0074; 125.0232
63′	Sebacic acid	10.72	C_10_H_18_O_4_	202.1205	201.1127	139.0025, 183.1019,59,3004
64′	Undecanedioic acid	11.79	C_11_H_20_O_4_	216.1361	215.1285	153.1285, 197.1184
65′	Dihexyl phthalate (isomers)	13.28/14.03/14.77/16.33	C_20_H_30_O_4_	334.2144	333.2074	150.0313, 251.0590, 151.0025
66′	13-keto-9Z,11E-octadecadienoic acid	16.40	C_18_H_28_O_3_	294.2194	293.2124	221.1282, 220.1291, 195.0913
67′	13S-hydroxy octadecadienoic acid	17.04	C_18_H_32_O_3_	296.2351	295.2279	96.9587, 195.1112; 277.1801
68′	Retinoic acid	18.32	C_20_H_28_O_2_	300.2090	299.2017	255.2118, 299.1918, 119.0769
69′	Icosanedioic acid/Eicosanedioic acid	19.02	C_20_H_38_O_4_	342.277	341.2707	98.9546, 84.0281
Other compounds						
70′	Syringin	7.86	C_17_H_24_ O_9_	372.1421	371.1348	194.0575, 209.0791, 311.1684
71′	Asystasioside A	8.06	C_22_H_34_O_14_	522.1949	521.1876	475.1822; 169.0497; 153.0547
72′	Sacranoside A	8.23	C_20_H_32_O_10_	432.1997	431.1924	205.1228; 153.0911; 89.0230
73′	Lucidumoside A	8.44	C_25_H_34_O_12_	526.205	525.1975	405.1191, 243.0659, 287.0561
74′	Diosbulbinoside F	8.68	C_26_H_34_O_12_	538.2050	537.1979	315.1241, 327.1240
75′	Denipride	8.69	C_18_H_26_N_4_O_5_	378.1891	377.1818	179.0705; 167.0704; 161.0598
76′	Propylparaben	9.07	C_10_H_12_O_3_	180.0786	179.0713	159.0441, 125.0231
77′	Enrasentan (isomers)	9.87/10.08/12.78	C_29_H_30_O_8_	506.1940	505.1867	163.0390, 243.0659, 505.1870
78′	Embelin	13.28/18.20	C_17_H_26_O_4_	294.1831	293.1761	293.1752, 184.0018
79′	Prohydrojasmon	14.64	C_15_H_26_O_3_	254.1882	253.1810	241.0477; 212.0477; 193.1591
80′	Furmecyclox	16.39	C_14_H_21_NO_3_	251.1521	250.1447	124.0152, 80.9637
81′	2-Ethylhexyl salicylate	16.40	C_12_H_26_O_4_S	266.1552	265.1480	96.9587, 79.9560, 95.9510
82′	Etienic acid	16.74	C_20_H_28_O_3_	316.2038	315.1966	124.8934, 78.9576
83′	Shogaol	17.50	C_17_H_24_O_3_	276.1725	275.1653	137.0232, 205.1594, 177.0909
84′	Myristyl sulfate	18.19	C_14_H_30_O_4_S	294.18669	293.1794	96.9586
85′	Abietic acid	19.08	C_20_H_30_O_2_	302.2245	301.2172	96.9587, 183.0113, 79.9559
86′	7-deoxyloganic acid glucopyranosyl ester	8.06	C_22_H_34_O_14_	522.1949	521.1876	315.1241, 327.1240

## Data Availability

Data available on request due to restrictions eg. privacy or ethical.

## Supplementary Materials

The following supporting information can be downloaded at: https://www.mdpi.com/article/10.3390/antiox12071383/s1, Figure S1: Pearson correlation between the bioactive characteristic and total phenolic acids and total flavonoids quantified by HRMS analysis in the coniferous extracts; Figure S2. The total ion current (TIC) chromatogram showing the identification of the main bioactive compounds in spruce needle extract using UHPLC–MS/MS detection in negative ionization mode; Figure S3. Total ion current (TIC) chromatogram showing the identification of the main bioactive compounds in fir bark extract using UHPLC–MS/MS detection in negative ionization mode; Figure S4. Total ion current (TIC) chromatogram showing the identification of the main bioactive compounds in fir needles extract using UHPLC–MS/MS detection in negative ionization mode; Table S1. The results of the quantitative estimation of the bioactive properties of the studied vegetal material, expressed in mg per g of dried weight vegetal material (range/mean ± SD); Table S2. Content of total polyphenols (TP), total flavonoids (TF), and antioxidant activity (AA) in coniferous biomass (bark and needles) according to data available in the literature; Table S3. Peak numbers, target compounds, expected retention times (tR), exact mass, accurate mass, mean mass accuracy (ppm), and resulted fragments (negative ionisation); Table S4. Phenolic compound content in coniferous biomass (bark and needles), according to data available in the literature; Table S5. Pharmacological activities of the bioactive compounds identified in spruce (*Picea abies* L., H. Karst) and fir (*Abies alba* Mill.) biomass (bark and needles) by non-target HRMS analysis; Table S6. The antimicrobial and anti-biofilm activities of *P. abies* and *A. alba* alcoholic extracts vs. solvent control; Table S7. Selectivity index values of *P. abies* bark and needle extracts and *A. abies* bark and needle extracts related to MIC and MBEC values; Table S8. Docking data for the best-ranked poses of the ligands in the two P–LD runs against PI3Kγ [1E8W entry] [55,56,57,58,73,74,75,76,95,96,97,98,99,100,101,102,103,104,105,120,121,122,124,126,127,128,129,130,134,135,138,139,145,162,163,164,165,166,167,168,169,170,171,172,173,174,175,176,177,178,179,180,181,182,183,184,185,186,187,188,189,190,191,192,193,194,195,196,197,198,199,200,201,202,203,204,205,206,207,208,209].
