# Supplementary material for: Antioxidant and Wound Healing Bioactive Potential of Extracts Obtained from Bark and Needles of Softwood Species
Source: Antioxidants (Basel). 2023 Jul 4;12(7):1383. doi: 10.3390/antiox12071383 (PMC10376860; doi:10.3390/antiox12071383)
Supplement: Supplementary file 1 [file antioxidants-12-01383-s001.zip › antioxidants-2475148-supplementary.pdf]

SUPPLEMENTARY MATERIAL

Wound healing bioactive potential of extracts obtained from bark and needles of softwood species

Elisabeta-Irina Geană[1,*], Corina Teodora Ciucure[1], Ioana Cristina Marinas[2,*], Diana Mădălina Gaboreanu[2,3], Miruna Stan[2], Carmen Lidia Chitescu[4]

[1] National R&D Institute for Cryogenics and Isotopic Technologies – ICSI Rm. Valcea, Romania
[2] Research Institute of the University of Bucharest-ICUB, Bucharest, Romania
[3] National Institute of Research and Development for Biological Sciences, 060031 Bucharest, Romania
[4] Faculty of Medicine and Pharmacy,"Dunarea de Jos" University of Galati, Galati, Romania
* Correspondence: irina.geana@icsi.ro; radu.tamaian@icsi.ro; cristina.marinas@icub.unibuc.ro

Supplementary Tables

Table S1. The results of the quantitative estimation of the bioactive properties of the studied vegetal material, expressed in mg per g of dried weight vegetal material (range / mean±SD)

**µg/g DW**	**spruce needles (n=13)**	**spruce bark (n=13)**	**fir needles (n=4)**	**fir bark (n=4)**
TP (mg GAE/g, dw)	54.31-107.06 / 77.03±14.29	22.92-141.53 / 105.83±31.00	44.50-80.37 / 58.00±15.52	21.64-40.28 / 30.21±9.01
TF (mg Q/g, dw)	2.53-22.43 / 8.94±6.76	1.46-23.24/ 9.91±7.46	2.76-3.67 / 3.17±0.38	1.24-2.81/ 2.01±0.86
AA (µMoli Trolox/g, dw)	220.26- 348.76 / 316.12±40.96	84.77-348.51 / 318.59±70.67	212.99-337.19 / 265.91±52.31	103.26-170.01 / 135.77±33.92

Table S2. Content of total polyphenols (TP), total flavonoids (TF) and antioxidant activity (AA) in coniferous biomass (bark and needles) according to the available literature data

Coniferous biomass	TP (mg GAE/g dw)	TF (mg Q/g dw)	AA (μM TE/g dw)
Pine bark	62.21 [1]<br>0.86 [2]<br>28.30 [3]<br>314.62 [4]<br>136.5 [5]	8.29 [2]<br>81.9 [5]	200.94 [2]<br>66.96 (mg TE/g dw) [4]
Spruce bark	13.30 [2]<br>54.97 [6]<br>404.0 [7]<br>130.26 [8]<br>426.0 [9]<br>11.03 [10]	3.54 [2]<br>14.44 [6]<br>53.4 [7]<br>39.91 [8]	404.18 [2]<br>308 [9]<br>128.4(mg TEs/g dw) [6]
Fir bark	14.53 [2]	7.46 [2]	269.55 [2]
Pine needles	14.00 [11]<br>274.38 [4]<br>7.6 [12]	7.20(mg CE/gextract) [11]<br>1.6 [12]	45.90(mg TE/g dw) [4]<br>305.5 [12]

Table S3. Peak numbers, target compounds, expected retention times (tR), exact mass, accurate mass, mean mass

No	Compound	Retention time [min]	Formula	Exact mass	Experimental Accurate mass [M-H]⁻	Mass fragments
Phenolic acids and derivatives						
**1**	Gallic acid	2.04	$C_7H_6O_5$	170.0215	169.0133	125.0231
**2**	3,4-Dihydroxybenzoic	4.22	$C_7H_6O_4$	154.0266	153.0182	109.0281
**3**	4-Hydroxybenzoic acid	6.45	$C_7H_6O_3$	138.0316	137.0231	93.0331
**4**	Chlorogenic acid	7.51	$C_{16}H_{18}O_9$	354.0950	353.0879	191.0552
**5**	Ferulic acid	7.78	$C_{10}H_{10}O_4$	194.0579	193.0499	178.0263, 134.0361
**6**	Syringic acid	8.04	$C_9H_{10}O_5$	198.0528	197.0448	182.0212, 153.0547, 138.0309
**7**	*p*-Coumaric acid	8.67	$C_9H_8O_3$	164.0473	163.0390	119.0489
**8**	Ellagic acid	9.91	$C_{14}H_6O_8$	302.0062	300.9990	300.9990
**9**	Abscisic acid	9.99	$C_{15}H_{20}O_4$	264.1361	263.1289	179.9803, 191.9454
**10**	Cinnamic acid	10.39	$C_9H_8O_2$	148.0524	147.0440	119.0487, 103.0387
**11**	Caffeic acid phenethyl ester (CAPE)	13.14	$C_{17}H_{16}O_4$	284.1048	283.0975	
Flavonoids						
**12**	(+)-Catechin	7.57	$C_{15}H_{14}O_6$	290.0790	289.0718	109.0278, 125.0228, 137.0232,
**13**	(-)-Epi-catechin	8.14	$C_{15}H_{14}O_6$	290.0790	289.0718	109.0278, 125.0228, 137.0232,
**14**	Myricetin	8.12	$C_{15}H_{10}O_8$	318.0375	317.0310	178.9986, 164.9263, 151.0036, 137.0244, 107.0125
**15**	Naringin	9.24	$C_{27}H_{32}O_{14}$	580.1791	579.1722	363.0722
**16**	Rutin	9.41	$C_{27}H_{30}O_{16}$	610.1533	609.1465	345.0616
**17**	Quercetin	10.71	$C_{15}H_{10}O_7$	302.0426	301.0355	151.0390, 178.9769, 121.0282
**18**	Kaempferol	11.65	$C_{15}H_{10}O_6$	286.0477	285.0406	151.0390, 117.0180
**19**	Isorhamnetin	11.82	$C_{16}H_{12}O_7$	316.0582	315.0512	300.0276
**20**	Apigenin	11.84	$C_{15}H_{10}O_5$	270.0528	269.0456	117.0180, 151.0390
**21**	Pinocembrin	12.52	$C_{15}H_{12}O_4$	256.0735	255.0661	213.0548, 151.0026, 107.0118
**22**	Chrysin	13.40	$C_{15}H_{10}O_4$	254.0579	253.0504	143.0491, 145.0494, 107.0118
**23**	Galangin	13.62	$C_{15}H10O_5$	270.0528	269.0454	169.0497, 143.0339
Stilbens						
**24**	*t*-Resveratrol	9.54	$C_{14}H_{12}O_3$	228.0786	227.0710	185.0811, 143.0339

accuracy (ppm) and resulted fragments (negative ionisation)

Table S4. Phenolic compounds content in coniferous biomass (bark and needles), according to the available literature data

**Phenolic compounds (μg/g dw)**	**Needles**			**Bark**		
	Picea abies	***Abies alba***	***Pinus sp.***	**Picea abies**	***Abies alba***	***Pinus sp.* (mg/L extract)**
Gallic acid	695.88 [2]	57.04 [2]	208.38 [2]	-	-	-
2,5-Dihydroxobenzoic Acid	41.3 [13]	5.4 [13]	14.1 [13]	-	-	
3,4-Dihydroxobenzoic Acid	-	-	-			29.7-64.1 [5]
4-Hydroxybenzoic Acid	-	--	-	-	-	
Vanillic acid	62.43 [2]	7.18 [2]	16.63 [2]	1.34 [14]	-	3.0-10.5 [5]
Syringic acid					-	
t-Cinnamic Acid	4014.44 [2]	1148.62 [2]	1084.92 [2]	4.73 [14]	-	5.4-53.4 [5]
Caffeic acid	227.0 [13]	38.8 [13]	55.8 [17]			4.2-13.8 [5]
Chlorogenic acid	1.56 [2]	0.95 [2]	0.46 [2]	-	--	5.8-17.2 [5]
p-Coumaric acid	7.6 [13]	194.4 [13]	7.1 [13]	-	-	-
Ferullic acid	-	113.97 [2]	145.44 [2]	-	-	9.7-24.5 [5]
Sinapinic Acid	301.96 [2]	4.3 [13]	8.9 [13]	2.94 [14]	-	
Salicylic acid	20.1 [13]	55.86 [2]	111.44 [2]	1.57 [15]	-	
Naringenin	819.74 [2]	29.8 [13]	8.7 [13]	-	-	
Catechin	45.1 [13]	1499.61 [2]	1502.03 [2]	11.17 [14]	-	105.0-135.0 [5]
Epicatechin	5094.84 [2]	30.8 [13]	107.0 [13]	24.39 [15]		-
Quercetin	287.9 [13]	984.09 [2]	518.25 [2]			8.5-10.9 [5]
Rutin	4534.29 [2]	10.9 [13]	29.1 [13]	0.51 [14]	-	-
Kaempferol	246.2 [13]	-	-	-	-	-
Apigenin	-	68.75 [2]	387.89 [2]		-	170.5 [8]
Luteolin	168.58 [2]	15.3 [13]	19.9 [13]	-	-	
Vitexin	9.0 [13]	-		-	-	
Taxifolin	-	502.20 [2]	2088.89 [2]		-	-
Resveratrol	1129.85 [2]	1.1 [13]	109.9 [13]	-	-	133.5 [8]
Proantocianidina B-2	70.2 [13]	-	-	-	-	

Table S5. Pharmacological activities of the bioactive compounds identified in spruce (*Picea abies* L., H. Karst.) and fir (*Abies alba* Mill.) biomass (bark and needles) by non -target HRMS analysis

No	Compound	PubChem CID	Pharmacological effects / Applications and medicinal activity	Spruce (*Picea abies*)		Fir (*Abies alba*)	
				Bark	Needles	Bark	Needles
**Phenolic acids and derivatives**							
**1′**	Quinic acid	6508	Antioxidant, antimicrobial, anti-inflammatory and neuroprotective [16]. A building block in the synthesis of Oseltamivir (Tamiflu), which is used to treat influenza A and B.	+	+	+	+
**2′**	Shikimic acid	8742	Anti-thrombotic, anti-inflammatory and bactericidal properties [17]. Is a common precursor of aromatic compounds produced in plants and microorganisms and is known as a raw material for producing the anti-influenza drug Tamiflu.	+	+	+	+
**3′**	5-Cffeoylquinic acid (Neochlorogenic acid)	5280633	Antioxidant and anti-inflammatory effects [18].	-	+	+	+
**4′**	Salicylic acid	338	Bacteriostatic, fungicidal, and keratolytic actions.	+	+	+	+
**5′**	Vanillic acid	8468	It have effective sedative, antioxidant, anti-inflammatory, and neuroprotective properties [19].	+	-	+	-
**6′**	Ferulic acid glucoside	24992652	It is a potential therapeutic candidate for prevention and treatment of cold stress injury and can significantly prevent cold-induced oxidative stress in liver and upregulate the protein level in brown fat tissue [20].	+	+	+	+
**7′**	3-p-coumaroylquinic acid/ izomeri	9945785	Is a primary metabolite. Is directly involved in an organism's growth, development, or reproduction (https://Hmdb.Ca).	-	+	+	+
**8′**	5-O-Caffeoylshikimic acid	5281762	Antioxidant, antimicrobial, anti-inflammatory, neuroprotective and antiproliferative activity against different cancer cell lines [21].	+	+	+	+
**9′**	Caffeic acid	689043	Immunomodulatory, anti-inflammatory, antimicrobial, antioxidant and antibacterial activity in vitro, and can contribute to the prevention of atherosclerosis and other cardiovascular diseases [22].	+	-	+	+
**10′**	Acetyl salicylic acid	2244	Non-steroidal anti-inflammatory minor analgesic, antipyretic, or anti-inflammatory. Low-dose aspirin has an antiplatelet effect and is used long-term to reduce the risk of heart attack (https://En.Wikipedia.Org).	+	+	+	+
**11′**	Vanillic acid glucoside	14132336	Antioxidant, anti-inflammatory [19].	+	+	+	+
**12′**	4-Methilbenzoic acid (toluic acid)	7470	It has a role as a xenobiotic metabolite (https://pubchem.ncbi.nlm.nih.gov/).	-	+	-	+
**13′**	Sinapic acid	637775	antioxidant, anti-cancer, anti-infammatory, cardioprotective, reno-protective, hepatoprotective, neuroprotective, anticarcinogenic, anti-diabetic and anti-bacterial properties [23].	-	+	+	+

**14′**	4-Methoxycinnamic acid	699414	Antioxidant and antilipidperoxidative effects, anti-inflammatory and anticancer properties of colon carcinogenesis [24].	+	+	+	+
**15′**	Coniferyl alcohol	1549095	It is an active ingredient in the products MULTI-PHENOLIC. Is indicated for relief of sensitivities due to allergic responses. May temporarily relieve symptoms associated with sensitivities to phenolic compounds, such as rash fatigue and lack of concentration (https://Pubchem.Ncbi.Nlm.Nih.Gov).	+	+	+	+
**16′**	Homovanilic acid	1738	Is a major catecholamine metabolite. It is used as a reagent to detect oxidative enzymes and is associated with dopamine levels in the brain (Https://Hmdb.Ca).	+	+	+	-
**Flavonoids and derivatives**							
**17′**	Epigallocatechin	72277	Antioxidant, a plant metabolite and a food component (Https://Foodb.Ca/).	+	+	+	+
**18′**	Procyanidin B1/B2	122738	Antioxidant, antibacterial, antineoplastic anti-obesity, anti-inflammatory and anti-allergic properties [25].				
**19′**	Taxifolin 3-O rhamnoside (isomers)	119258	Analgesic and anti-edema effect (Www.Lifeasible.Com).	+	+	+	+
**20′**	Phloretin	4788	Antioxidant and antidiabetic properties. It has a role as a plant metabolite and an antineoplastic agent. It can be used in facial masks, skin creams, lotions and essences (Https://En.Wikipedia.Org).	+	+	+	+
**21′**	Dihydrokaempferol (aromodendrin)	662	Anti-inflammatory, antioxidant, anti-diabetic [26].	+	+	+	+
**22′**	Taxifolin glucoside	14187089	Porcine pancreatic lipase inhibitory activity, antioxidant activity [27].	+	+	+	+
**23′**	Phloretin - 2-O glucoside	46926173	Estrogenic and antiestrogenic activities. It is used in the control of blood sugar levels and as a protective agent to the heart from scavenging oxygen free radicals and antioxidant injuries [28] (Https://Pubchem.Ncbi.Nlm.Nih.Gov).	+	+	+	+
**24′**	Proanthocyanidin	108065	Antioxidant, antitumor, cardioprotector, neuroprotector, antidiabetic, antimicrobial and immunostimulating properties [28].	+	+	+	+
**25′**	Procyanidin A2	6325839	Antioxidant, antibacterial, antineoplastic anti-obesity, anti-inflammatory and anti-allergic properties [25].	+	+	+	+
**26′**	Tricin	5281702	Anti-inflammatory and is role as a chemopreventive and anticancer agent (Https://Natuprod.Bocsci.Com).	-	+	+	+
**27′**	Taxifolin	439533	Antioxidant, anti-inflammation, antiviral, antitumor, antibacterial, and enzyme inhibition [27].	+	+	+	+
**28′**	Vitexin-2-O-rhamnoside	5282151	Supports the degradation of oxidative stress and has the potential to treat diseases of the cardiovascular system (Https://Www.Medchemexpress.Com).	-	+	-	++
**29′**	Cedeodarin	182026	Anti-inflammatory, anti-bacterial, and anti-fungal properties. to inhibit the growth of various types of cancer cells (Https://Www.Benchchem.Com).	+	+	+	+

**30´**	Myricetin 3 O rhamnoside	5352000	Antibacterial, anti-inflammatory, antioxidant, antitumor promoter, diuretic [29].	+	+	+	+
**31´**	kaempferol-3- O- rutinoside	122173234	Hypoglycemic effect, biological and anti-obesity activity [30].	+	+	+	+
**32´**	Kaempferol/luteolin-O-glucoside/ isomers	5319116	Anti-inflammatory, antioxidant, anti-hyperuricemia and anti-gout effects [31].	+	+	+	+
**33´**	vitexin (apigenin 8- C-glucoside)/isovitexin	5280441	Anti-cancer, anti-oxidant, anti-inflammatory, anti-nociceptive, anti-AD (AD, Alzheimer's disease), anti-hypertensive, anti-spasmodic, anti-hypoxia/ischemia injury, anti-depressant-like actions and anti-viral activities [32]	+	+	+	+++
**34´**	Pratensein/Tectprogenin	5281803	can have effects for the prevention of atherosclerosis (Https://En.Wikipedia.Org.)	+	+	+	+
**35´**	Afrormosin	5281704	anti-inflammatory, antioxidant and immunomodulatory properties [33]	+	+	+	+
**36´**	Liquiritigenin/liquiritigenin	114829	Antispasmodic, antiulcer, antibacterial, hepatocyte monoamine oxidase inhibitor [34].	+	+	+	+
**37´**	Pinostrobin	4101463	antioxidant, analgesic, anti-hemorrhagic and dermal anti-inflammatory [35]	+	+	+	+
**38´**	Glycitein / biochanin A	5317750	Estrogenic, antioxidant, hypocholesterolemic and cell proliferation inhibitory activities [36].	+	+	+	+
**39′**	Daidzein	5281708	Estrogenic, antioxidant, hypocholesterolemic and cell proliferation inhibitory activities [37]. It has a role as an antineoplastic agent, a phytoestrogen, a plant metabolite.	+	+	+	+
**40´**	3-Methyl galangin	5281946	Antibacterial activities which also inhibits pancreatic lipase [38]	+	+	+	+
**Stilbenes and derivatives**							
**41´**	Astringin	5281712	Antibacterial, antifungal, antitermite, anticancer, antioxidant [39]	+	+	+	+
**42´**	Piceatannol glucoside	11968990	Anti-inflammatory, antioxidant, and anti-cancer activities. In addition, it has been shown to have anti-diabetic, anti-bacterial, and anti-viral activities (Https://En.Wikipedia.Org).	+	+	+	+
**43´**	*t*-Piceid	5281718	Antithermic [39].	+	+	+	+
**44´**	Combretastatin A-4 (isomers)	5351344	Anticancer, antioxidant [40].	+	+	+	+
**45´**	*t*-Isorhapontin	5281716	Antibacterial, antifungal, antithermic [39].	+	+	+	+
**46´**	*t*-Isorhapontigenin	5318650	Antibacterial, antifungal, antithermic, cardioprotection, antioxidant [39].	+	+	+	-
**47´**	Piceatannol	667639	Antibacterial, cardioprotection, anticancer, antioxidant [39].	+	+	+	+
**Terpenes**							
**48´**	Toosendanin (isomers)	115060	Analgesic, insecticidal and anti-inflammatory activities [41].	+	+	+	+
**49´**	Vernodalin	179375	The active compound responsible for the anti-cancer property in human breast cancer [42].	+	+	+	+
**50´**	Inulicin (isomers)	75528891	Inhibits angiogenesis and lung cancer cell growth [43].	+	+	+	+
**51´**	Podocarpic acid	93017	Antioxidative,anti-inflammatory, anticancer [44].	-	-	+	+
**52´**	Carnosol (isomers)	442009	Antioxidative, anti-inflammatory, antiproliferative [45]	+	+	+	+

**53′**	Carnosic acid (isomers)	65126	Antimicrobial, antioxidant and protection against carcinogens [45].	+	+	+	+
**54′**	Rosmaridiphenol/izomer	9905016	Antioxidant, cancer preventive [46].	+	+	+	+
**55′**	Rosmanol methyl ether	101611746	Anti-inflammatory, anti-angiogenic, anti-carcinogenic, neuroprotective, and anti-obesity effects (Https://Www.Benchchem.Com).	++	-	++	+
**Lignans and derivatives**							
**56′**	Hydroxymatairesinol (isomers)	10948757	Anti-oxidant and it is an enterolactone precursor with anticancer activities (Https://En.Wikipedia.Org).	+	+	+	+
**57′**	(+)-Pinoresinol	73399	Anticancer, hypoglycemic and antifungal agent [47].	+	+	+	+
**58′**	Sesquipinsapol B	101767126	Antioxidant, cytotoxic [48].	-	+	+	+
**59′**	Lariciıesinol / isolariciresinol	160521	Antioxidant; anti-inflammatory [37].	+	+	+	+
**Carboxilic acids and derivatives**							
**60′**	Citric acid	311	Anti-microbial, anti-inflammatory, anti-cancer, anti-inflammatory and anti-cancer [49].	+	+	+	+
**61′**	Suberic acid	10457	Energy storage. Is used in the preparation of reduction-sensitive micelles affecting their cellular uptake. This has potential application in delivery of anticancer drugs [50].	+	+	+	+
**62′**	Azelaic acid	2266	Antibacterial, antimicrobial, anti-inflammatory, antineoplastic agent, a dermatologic drug and a plant metabolite [51].	+	+	+	+
**63′**	Sebacic acid	5192	Anti-HIV activity [52]	+	+	+	+
**64′**	Undecanedioic acid	15816	Nutrient with biological role is membrane stabilizer [53].	+	+	+	+
**65′**	Dihexyl phthalate (isomers)	6786	No available study on bioactivity.	+	+	+	+
**66′**	13-keto-9Z,11E-octadecadienoic acid	6446027	stimulate cell proliferation from colonic mucosa [53].	+	+	+	+
**67′**	13S-hydroxy octadecadienoic acid	6443013	Anticancer and biomarker from autosomal dominant polycystic kidney disease (Https://Hmdb.Ca).	+	+	+	+
**68′**	Retinoic acid	11130378	Is essential for development of many organs including the hindbrain, spinal cord, forelimb buds, skeleton, heart, eye, pancreas, lung and genitourinary tract [54].	+	+	+	+
**69′**	Icosanedioic acid / Eicosanedioic acid	75502	Uses are often used in cosmetics and used as emulsifiers. Belongs to the technical field of daily chemical skin care products [55].	+	+	+	+
**Other compounds**							
**70′**	Syringin	5316860	It has a role as a hepatoprotective agent, antidiabetic effects and significant anti-inflammatory activity [56].	+	+	+	+
**71′**	Asystasioside A	101831547	Hypoglycemic agent with potential α-glucosidase inhibitory activities (https://pubchem.ncbi.nlm.nih.gov/).	+	+	+	+

**72′**	Sacranoside A	102094959	the anti-HCV activity, drug candidate for antiviral activity against HCV (https://pubchem.ncbi.nlm.nih.gov/).	+	+	+	+
**73′**	Lucidumoside A	637081	antioxidant activity against hemolysis of red blood cells induced by free radicals.; antiviral agent [57]	+	+	+	+
**74′**	Diosbulbinoside F	181842	Compound identified in the rhizome of D. bulbifera which has been used in folk and traditional Chinese medicine for the treatment of thyroid diseases and cancer [53][58]	+	+	+	+
**75′**	Denipride	205974	has a positive impact on the central nervous system and has been studied for its potential use in treating neurological disorders such as Parkinson's disease (Https://Pubchem.Ncbi.Nlm.Nih.Gov.)	+	+	+	+
**76′**	Propylparaben	7175	Antifungal and antimicrobial properties. is typically used in a variety of water-based cosmetics and personal-care products (Https://En.Wikipedia.Org.)	+	+	+	+
**77′**	Enrasentan (isomers)	178103	It has a role as an endothelin receptor antagonist and an antihypertensive agent (Https://Pubchem.Ncbi.Nlm.Nih.Gov.)	+	+	+	+
**78′**	Embelin	3218	It exhibits antimicrobial, antineoplastic and inhibitory activity towards hepatitis C protease. It has a role as a hepatitis C protease inhibitor, an antimicrobial agent, an antineoplastic agent and a plant metabolite (Https://Pubchem.Ncbi.Nlm.Nih.Gov.)	+	+	+	+
**79′**	Prohydrojasmon		No available study on bioactivity				[54]
**80′**	Furmecyclox	43359	Furamide fungicide (Https://Pubchem.Ncbi.Nlm.Nih.Gov).	+	+	+	+
**81′**	2-Ethylhexyl salicylate		The salicylate portion of the molecule absorbs ultraviolet light to protect skin from the harmful effects of exposure to sunlight, while the ethylhexanol portion functions as an emollient (https://pubchem.ncbi.nlm.nih.gov/)				
**82′**	Etienic acid	99472	Anticancer, anti-inflammatory, anticoagulant, antimicrobial, insecticidal/pesticidal, antioxidant and antiviral activities [59].	+	+	+	+
**83′**	Shogaol	5281794	6-Shogaol exhibits a variety of biological activities including anticancer, anti-inflammation, and anti-oxidation. It has a very strong antitussive (anti-cough effect) [60].	+	+	+	+
**84′**	Myristyl sulfate	5248	Sclerosing agent. It is commonly used in the treatment of small varicose veins of the legs, as well as venous and lymphatic malformations [61].	+	+	+	+
**85′**	Abietic acid	10569	Antimicrobial, anti-inflammatory, antioxidant, and anticancer properties [62].	+	+	+	+
**86′**	7-deoxyloganic acid glucopyranosyl ester	28285160*	Is a 7-deoxyloganic acid derivative (iridoid glycoside), which is a plant metabolite with anti-inflammatory effects by inhibiting the production of pro-inflammatory cytokines and antioxidant effects by scavenging free radicals and reducing oxidative stress [63].			+	

Table S6. The antimicrobial and anti-biofilm activities of *P. abies* and *A. alba* alcoholic extracts vs. solvent control

		***P. aeruginosa* ATCC 27853**	***S. aureus* sc pl**	***E. faecalis* ATCC 19433**	**MRSA**	***S. marcescens* Ec5K**	***C. albicans* ATCC 10231**
**Fir bark extract**	MIC (μL/mL)	31.25	250	125	125	62.5	250
	MBEC (μL/mL)	31.25	125	125	15.625	62.5	15.625
**Spruce needles extract**	MIC (μL/mL)	31.25	125	250	125	31.25	62.5
	MBEC (μL/mL)	31.25	31.25	250	125	31.25	31.25
**Fir needles extract**	MIC (μL/mL)	15.625	31.25	125	62.5	62.5	62.5
	MBEC (μL/mL)	15.625	15.625	125	62.5	62.5	31.25
**Spruce bark extract**	MIC (μL/mL)	31.25	62.5	125	62.5	62.5	500
	MBEC (μL/mL)	31.25	62.5	125	62.5	62.5	31.25
**EtOH 50%**	MIC (μL/mL)	62.5	250	125	250	62.5	250
	MBEC (μL/mL)	31.25	250	62.5	250	62.5	125

Table S7. Selectivity index values of *P. abies* bark and needles extracts and *A. abies* bark and needles extracts related to MIC and MBEC values

		***P. aeruginosa* ATCC 27853**	***Interp.***	***S. aureus* sc pl**	***Interp.***	***E. faecalis* ATCC 19433**	***Interp.***	**MRSA**	***Interp.***	***S. marcescens* Ec5K**	***Interp.***	***C. albicans* ATCC 10231**	***Interp.***
**Fir bark extract**	MIC	1.31	**B	0.16	*A	0.32	*A	0.33	*A	0.65	*A	0.16	*A
	MBEC	1.31	**B	0.16	*A	0.32	*A	2.62	**B	0.65	*A	2.62	**B
**Spruce needles extract**	MIC	1.15	**B	0.14	*A	0.14	*A	0.29	*A	0.58	*A	0.58	*A
	MBEC	1.15	**B	1.14	**B	0.14	*A	0.29	*A	1.15	**B	1.15	**B
**Fir needles extract**	MIC	1.17	**B	0.58	*A	0.14	*A	0.29	*A	0.29	*A	0.59	*A
	MBEC	1.17	**B	1.17	**B	0.14	*A	0.29	*A	0.29	*A	0.59	*A
**Spruce bark extract**	MIC	0.76	*A	0.38	*A	0.19	*A	0.38	*A	0.38	*A	0.05	*A
	MBEC	0.76	*A	0.38	*A	0.19	*A	0.38	*A	0.38	*A	0.76	*A

*A - more toxic to eukaryotic cell; **B—more toxic to microbial cell

Table S8. Docking data for the best ranked poses of the ligands in the two P–LD runs against PI3Kγ [1E8W entry]

Target – Ligand complex	PyRx w/ Autodock Vina	SwissDock w/ EADock DSS		Notes
	BA (kcal/mol)	*FullFitness* (kcal/mol)	ΔG (kcal/mol)	
1E8W – 13-Keto-9Z,11E-octadecadienoic acid	-6.7	-4612.05	-8.61	Qualitative analysis
1E8W – 13S-Hydroxyoctadecadienoic acid	-6.9	-4622.29	-7.52	Qualitative analysis
1E8W – 3,4-Dihydroxybenzoic acid	-6.5	-4572.04	-6.53	Quantitative data
1E8W – 4-Hydroxybenzoic acid	-6.1	-4574.26	-6.39	Quantitative data
1E8W – 4-Methoxycinnamic acid	-7.2	-4581.41	-6.91	Qualitative analysis
1E8W – Abscisic acid	-7.0	-4584.30	-7.21	Quantitative data
1E8W – Apigenin	-8.7	-4574.08	-7.22	Quantitative data
1E8W – Astringin	-8.9	-4505.15	-8.36	Qualitative analysis
1E8W – Azelaic acid	-6.4	-4625.59	-7.30	Qualitative analysis
1E8W – Caffeic acid phenethyl ester (CAPE)	-8.0	4564.93	-8.03	Quantitative data
1E8W – Chlorogenic acid	-8.3	4537.02	-8.71	Quantitative data
1E8W – Chrysin	-8.8	-4564.64	-7.10	Quantitative data
1E8W – Cinnamic acid	-7.0	-4582.21	-6.49	Quantitative data
1E8W – Citric acid	-5.6	-4619.89	-6.35	Qualitative analysis
1E8W – Combretastatin A-4	-7.1	-4520.50	-7.83	Qualitative analysis
1E8W – Ellagic acid	-9.4	-4521.51	-7.96	Quantitative data
1E8W – Embelin	-6.9	-4565.69	-7.56	Qualitative analysis
1E8W – Enrasentan	-9.1	-4560.13	-8.34	Qualitative analysis
1E8W – Epigallocatechin	-8.3	-4559.63	-8.09	Qualitative analysis
1E8W – Ferulic acid	-7.2	-4582.86	-7.08	Quantitative data
1E8W – Furmecyclox	-6.8	-4563.49	-7.27	Qualitative analysis
1E8W – Galangin	-8.8	-4545.43	-7.30	Quantitative data
1E8W – Gallic acid	-6.6	-4571.72	-6.55	Quantitative data
1E8W – Icosanedioic acid	-6.8	-4646.61	-8.37	Qualitative analysis
1E8W – Isorhamnetin	-9.0	-4541.74	-7.98	Quantitative data
1E8W – Isorhapontigenin	-7.9	-4575.25	-7.60	Qualitative analysis
1E8W – Isorhapontin	-8.8	-4490.86	-8.24	Qualitative analysis
1E8W – Kaempferol	-8.7	-4560.14	-8.05	Quantitative data
1E8W – Lucidumoside A	-8.9	-4530.20	-8.98	Qualitative analysis
1E8W – Methylbenzoic acid	-6.2	-4561.45	-6.40	Qualitative analysis
1E8W – Myricetin	-9.0	-4551.94	-8.40	Quantitative data
1E8W – Myristyl sulfate	-6.2	-4673.09	-8.54	Qualitative analysis
1E8W – Naringin	-9.5	-4444.41	-9.92	Quantitative data
1E8W – Oxododecanedioic acid	-7.2	-4637.53	-7.99	Qualitative analysis
1E8W – p-Coumaric acid	-7.0	-4593.66	-6.78	Quantitative data
1E8W – Pinocembrin	-8.8	-4577.38	-7.03	Quantitative data
1E8W – Prohydrojasmon	-6.6	4594.38	-7.52	Qualitative analysis
1E8W – Propylparaben	-6.7	-4569.85	-6.78	Qualitative analysis

**1E8W – Quercetin [RE-DOCKING]**	**-9.1**	**-4552.31**	**-7.68**	Quantitative data
1E8W – Quinic acid	-5.9	-4534.49	-6.60	Qualitative analysis
1E8W – Resveratrol	-7.9	-4587.38	-7.09	Quantitative data
1E8W – Rutin	-9.5	-4418.05	-10.16	Quantitative data
1E8W – Sebacic acid	-6.5	-4629.80	-7.38	Qualitative analysis
1E8W – Shikimic acid	-5.9	-4558.70	-6.21	Qualitative analysis
1E8W – Shogaol	-7.5	-4570.36	-7.91	Qualitative analysis
1E8W – Suberic acid	-6.3	-4628.21	-7.46	Qualitative analysis
1E8W – Syringic acid	-6.6	-4552.23	-7.04	Quantitative data
1E8W – Taxifolin	-9.2	-4552.84	-7.58	Qualitative analysis
1E8W – Toosendanin	-8.3	-4293.84	-8.15	Qualitative analysis
1E8W – Undecanedioic acid	-6.7	-4629.87	-8.10	Qualitative analysis
1E8W – (+)-Catechin	-8.8	-4564.29	-7.92	Quantitative data
1E8W – (-)-Epicatechin	-8.4	-4558.15	-6.93	Quantitative data
1E8W – (+)-Pinoresinol	-7.8	-4523.94	-7.86	Qualitative analysis

Supplementary Figures

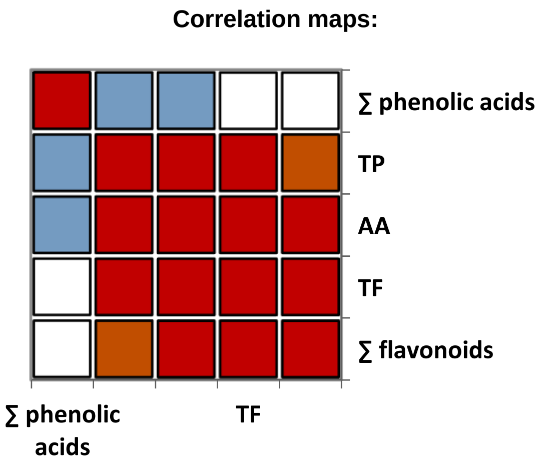

Correlation matrix (Pearson):

Variables	∑ Phenolic acids	TP	AA	TF	∑ Flavonoids
∑ Phenolic acids	**1**	-0.293	-0.361	0.067	0.001
TP	-0.293	**1**	**0.899**	**0.927**	0.694
AA	-0.361	**0.899**	**1**	**0.850**	**0.895**
TF	0.067	**0.927**	**0.850**	**1**	**0.806**
∑ Flavonoids	0.001	0.694	**0.895**	**0.806**	**1**

Values in bold are different from 0 with a significance level alpha=0.05

Figure S1. Pearson correlation between the bioactive characteristic and total phenolic acids and total flavonoids quantified by HRMS analysis in the coniferous extracts

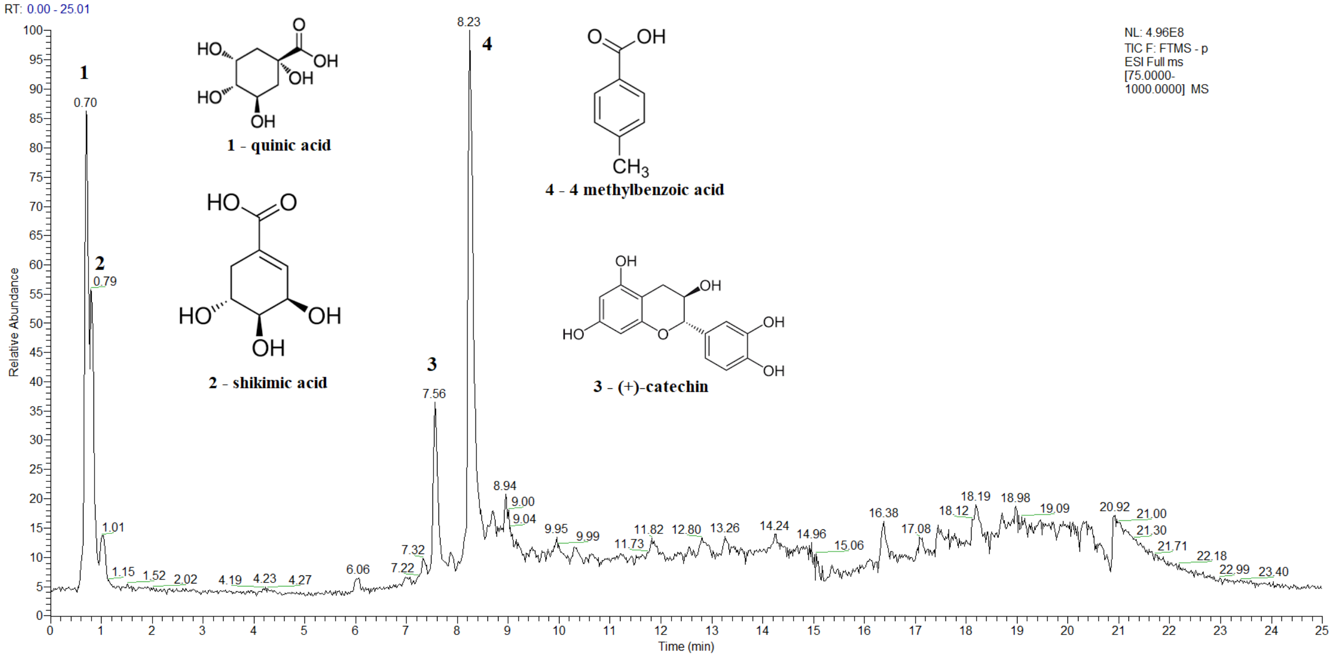

Figure S2. The total ion current (TIC) chromatogram showing the identification of the main bioactive compounds in spruce needles extract using UHPLC–MS/MS detection in negative ionization mode

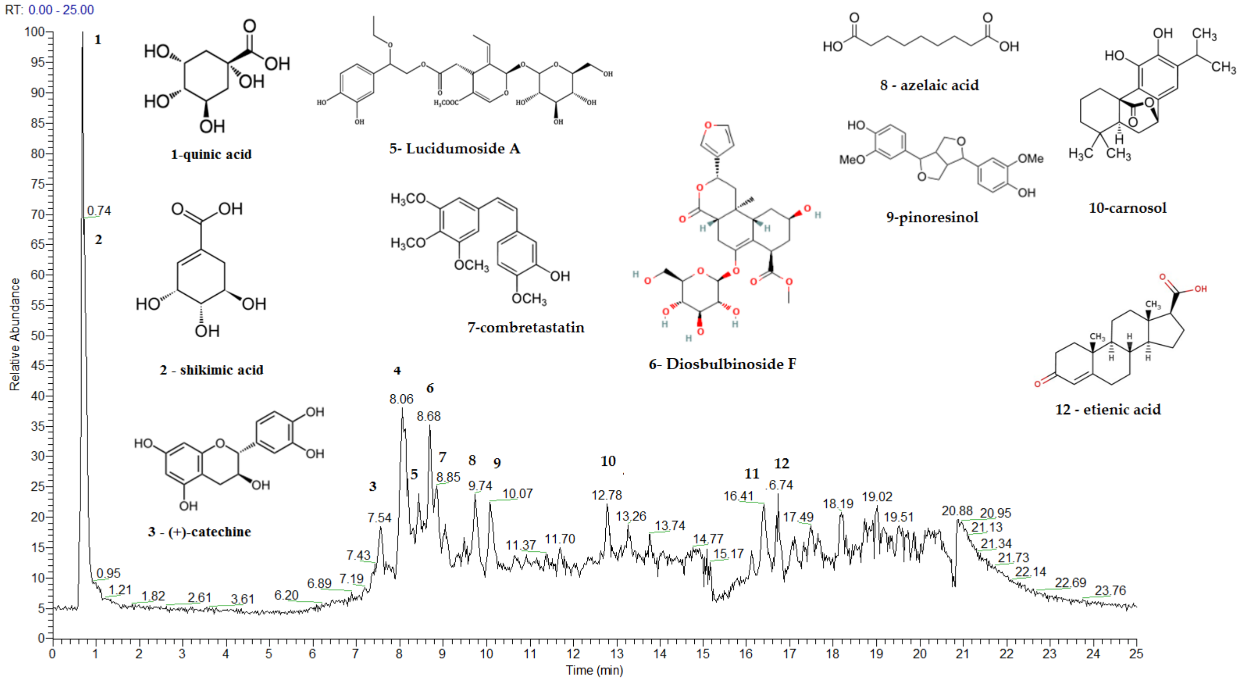

Figure S3. The total ion current (TIC) chromatogram showing the identification of the main bioactive compounds in fir bark extract using UHPLC–MS/MS detection in negative ionization mode

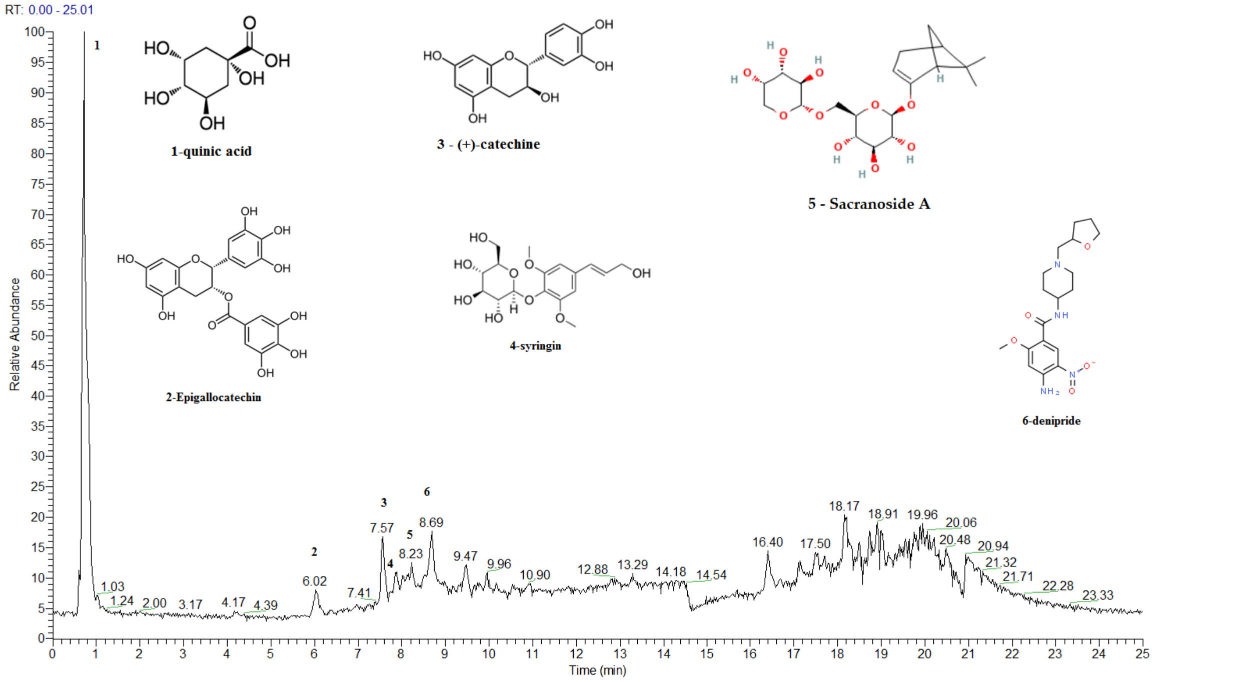

Figure S4. The total ion current (TIC) chromatogram showing the identification of the main bioactive compounds in fir needles extract using UHPLC–MS/MS detection in negative ionization mode

Materials and methods

Protein – Ligand Docking (P–LD)

For the *Protein – Ligand Docking (P–LD)* study it was chose another RCSB PDB entry for PI3Kγ, namely 1E8W due its better resolution (2.50 Å) [64]. Moreover, the co-crystallized ligands from the two RCSB PDB entries are two of the quantitatively identified natural compounds in our extracts: quercetin (ligand of 1E8W – binding affinity data: IC_{50} = 3.80E+3nM and K_d = 280nM) [64] and myricetin (ligand of 1E90 – binding affinity data: IC_{50} = 1.80E+3nM and K_d = 170nM) [64].

As a pre-docking operation, the structural files (PDB file format) for the two entries (1E8W and 1E90) [64]were downloaded from RCSB PDB [65–67] and superimposed with Molegro Molecular Viewer 2019 7.0.0 (MMV7 – Molexus IVS, Odder, Denmark).

Structure-data files (SDF file format) for ligands (the quantitative identified phenolics and the majority phenolic compounds) were obtained from PubChem (https://pubchem.ncbi.nlm.nih.gov) [68], then individually optimized and converted in Tripos MOL2 file format (MOL2), as required for docking. MarvinSketch was used for tridimensional (3D) optimization and generations of ligands files, MarvinSketch version 23.8.0, 2023, ChemAxon (https://chemaxon.com/).

Two comparative P–LD runs were performed against the selected molecular target enzyme (RCSB PDB ID: 1E8W) with two different software by using two different docking algorithms (and different scoring function used to approximate the standard chemical potentials of the system) for cross-validation of results. Both P–LD runs were performed within the same search space around the binding region of quercetin (the co-crystallized ligand from the 1E8W RCSB PDB entry) to improve the docking accuracy: center coordinates: X = 25.570; Y = 50.836; Z = 26.578; dimensions (Å): X = 21.323; Y = 32.008; Z = 25.000.

First P–LD run was performed on local cloud with the AutoDock Vina v.1.2.0 algorithm [68,69] using PyRx—Python Prescription v.0.9.7 software [70] as user interface (UI) control. AutoDock Vina is a flexible-ligand docking algorithm [71], which automatically calculates the grid maps and clusters the docking results in a user accessible way for an improved user experience (UX) [68,69]. The search algorithm implemented in AutoDock Vina is a Monte–Carlo iterated search algorithm [72] combined with the Broyden–Fletcher–Goldfarb–Shanno (BFGS) gradient-based optimizer (an iterative method for solving unconstrained nonlinear optimization problems) [73,74]. Scoring function used by AutoDock Vina combines the knowledge-based potentials with empirical scoring functions and it was inspired by X-score [75] and derived with the help of the PDBbind data set [76,77]. Ranking of poses is made by measuring the distance between the experimental and the predicted structures, expressed as RMSD (root-mean-square deviation), with two terms: RMSD lower bound (RMSD/lb) and RMSD upper bound (RMSD/ub). In our docking set-up, the value of exhaustiveness (the amount of computational time spent during a docking run) was set to 200 to improve the consistency of the docking results (the scoring function requires higher exhaustiveness values for achieving good performances, while the preset value of exhaustiveness is 8) [69], disregarding the increasing computational time.

The second P–LD run was performed with the help of the SwissDock web-service (http://www.swissdock.ch/) [78], using EADock DSS algorithm [78,79]. EADock DSS is a flexible-ligand docking algorithm [71] with an innovative and improved sampling engine (dihedral space sampling – DDS). EADock DSS benefits of a high accuracy of a multi-objective evolutionary algorithm [80] combining two scoring functions based on the CHARMM22 force field developed for the CHARMM software [81]. Ranking of poses is made bases on the *FullFitness* term, which accounts for the solvation free energy using the Generalized Born model implemented in CHARMM [81]. In our docking set-up, the docking type was set to "accurate" from the SwissDock web-service interface.

The co-crystallized ligand of PI3Kγ/1E8W (quercetin) was re-docked, serving a reference or control molecule for both P–LD runs.

References:

1. Chupin, L.; Motillon, C.; Charrier-El Bouhtoury, F.; Pizzi, A.; Charrier, B. Characterisation of Maritime Pine (Pinus Pinaster) Bark Tannins Extracted under Different Conditions by Spectroscopic Methods, FTIR and HPLC. *Industrial Crops and Products* **2013**, *49*, 897–903, doi:10.1016/J.INDCROP.2013.06.045.
2. Dziedzinski, M.; Kobus-Cisowska, J.; Szymanowska, D.; Stuper-Szablewska, K.; Baranowska, M. Identification of Polyphenols from Coniferous Shoots as Natural Antioxidants and Antimicrobial Compounds. *Molecules 2020, Vol. 25, Page 3527* **2020**, *25*, 3527, doi:10.3390/MOLECULES25153527.
3. Chupin, L.; Maunu, S.L.; Reynaud, S.; Pizzi, A.; Charrier, B.; Charrier-EL Bouhtoury, F. Microwave Assisted Extraction of Maritime Pine (Pinus Pinaster) Bark: Impact of Particle Size and Characterization. *Industrial Crops and Products* **2015**, *65*, 142–149, doi:10.1016/j.indcrop.2014.11.052.
4. Duarte, H.; Gomes, V.; Aliaño-González, M.J.; Faleiro, L.; Romano, A.; Medronho, B. Ultrasound-Assisted Extraction of Polyphenols from Maritime Pine Residues with Deep Eutectic Solvents. *Foods 2022, Vol. 11, Page 3754* **2022**, *11*, 3754, doi:10.3390/FOODS11233754.
5. Ferreira-Santos, P.; Genisheva, Z.; Botelho, C.; Santos, J.; Ramos, C.; Teixeira, J.A.; Rocha, C.M.R. Unravelling the Biological Potential of Pinus Pinaster Bark Extracts. *Antioxidants 2020, Vol. 9, Page 334* **2020**, *9*, 334, doi:10.3390/ANTIOX9040334.
6. Spinelli, S.; Costa, C.; Conte, A.; Porta, N.L.; Padalino, L.; Del Nobile, M.A. Bioactive Compounds from Norway Spruce Bark: Comparison Among Sustainable Extraction Techniques for Potential Food Applications. *Foods 2019, Vol. 8, Page 524* **2019**, *8*, 524, doi:10.3390/FOODS8110524.
7. García-Pérez, M.E.; Royer, M.; Herbette, G.; Desjardins, Y.; Pouliot, R.; Stevanovic, T. Picea Mariana Bark: A New Source of Trans-Resveratrol and Other Bioactive Polyphenols. *Food Chemistry* **2012**, *135*, 1173–1182, doi:10.1016/J.FOODCHEM.2012.05.050.
8. Talmaciu, A.I.; Ravber, M.; Volf, I.; Knez, Ž.; Popa, V.I. Isolation of Bioactive Compounds from Spruce Bark Waste Using Sub- and Supercritical Fluids. *The Journal of Supercritical Fluids* **2016**, *117*, 243–251, doi:10.1016/J.SUPFLU.2016.07.001.
9. Francezon, N.; Stevanovic, T. Integrated Process for the Production of Natural Extracts from Black Spruce Bark. *Industrial Crops and Products* **2017**, *108*, 348–354, doi:10.1016/J.INDCROP.2017.06.052.
10. Strižincová, P.; Ház, A.; Burčová, Z.; Feranc, J.; Kreps, F.; Šurina, I.; Jablonský, M. Spruce Bark—A Source of Polyphenolic Compounds: Optimizing the Operating Conditions of Supercritical Carbon Dioxide Extraction. *Molecules* **2019**, *24*, doi:10.3390/MOLECULES24224049.
11. Karapandzova, M.; Stefkov, G.; Cvetkovikj, I.; Stanoeva, J.P.; Stefova, M.; Kulevanova, S. Flavonoids and Other Phenolic Compounds in Needles of Pinus Peuce and Other Pine Species from the Macedonian Flora. *https://doi.org/10.1177/1934578X1501000647* **2015**, *10*, doi:10.1177/1934578X1501000647.
12. Yen, G.C.; Duh, P.D.; Huang, D.W.; Hsu, C.L.; Fu, T.Y.C. Protective Effect of Pine (Pinus Morrisonicola Hay.) Needle on LDL Oxidation and Its Anti-Inflammatory Action by Modulation of INOS and COX-2 Expression in LPS-Stimulated RAW 264.7 Macrophages. *Food and Chemical Toxicology* **2008**, *46*, 175–185, doi:10.1016/J.FCT.2007.07.012.
13. Szwajkowska-Michałek, L.; Przybylska-Balcerek, A.; Rogoziński, T.; Stuper-Szablewska, K. Phenolic Compounds in Trees and Shrubs of Central Europe. *Applied Sciences 2020, Vol. 10, Page 6907* **2020**, *10*, 6907, doi:10.3390/APP10196907.
14. Chmelová, D.; Škulcová, D.; Legerská, B.; Horník, M.; Ondrejovič, M. Ultrasonic-Assisted Extraction of Polyphenols and Antioxidants from Picea Abies Bark. *Journal of Biotechnology* **2020**, *314–315*, 25–33, doi:10.1016/J.JBIOTEC.2020.04.003.

15. Bocalandro, C.; Sanhueza, V.; Gómez-Caravaca, A.M.; González-álvarez, J.; Fernández, K.; Roeckel, M.; Rodríguez-Estrada, M.T. Comparison of the Composition of Pinus Radiata Bark Extracts Obtained at Bench- and Pilot-Scales. *Industrial Crops and Products* **2012**, *38*, 21–26, doi:10.1016/J.INDCROP.2012.01.001.
16. Bai, J.; Wu, Y.; Zhong, K.; Xiao, K.; Liu, L.; Huang, Y.; Wang, Z.; Hong, G.A.O. A Comparative Study on the Effects of Quinic Acid and Shikimic Acid on Cellular Functions of Staphylococcus Aureus. *Journal of Food Protection* **2018**, *81*, 1187–1192, doi:10.4315/0362-028X.JFP-18-014.
17. Wu, S.; Chen, W.; Lu, S.; Zhang, H.; Yin, L. Metabolic Engineering of Shikimic Acid Biosynthesis Pathway for the Production of Shikimic Acid and Its Branched Products in Microorganisms: Advances and Prospects. *Molecules 2022, Vol. 27, Page 4779* **2022**, *27*, 4779, doi:10.3390/MOLECULES27154779.
18. Gao, X. han; Zhang, S. dong; Wang, L. tao; Yu, L.; Zhao, X. lian; Ni, H. yan; Wang, Y. qiu; Wang, J. dong; Shan, C. hua; Fu, Y. jie Anti-Inflammatory Effects of Neochlorogenic Acid Extract from Mulberry Leaf (Morus Alba L.) Against LPS-Stimulated Inflammatory Response through Mediating the AMPK/Nrf2 Signaling Pathway in A549 Cells. *Molecules 2020, Vol. 25, Page 1385* **2020**, *25*, 1385, doi:10.3390/MOLECULES25061385.
19. Ingole, A.; Kadam, M.P.; Dalu, A.P.; Kute, S.M.; Mange, P.R.; Theng, V.D.; Lahane, O.R.; Nikas, A.P.; Kawal, Y.V.; Nagrik, S.U.; et al. A Review of the Pharmacological Characteristics of Vanillic Acid. *J. Drug Delivery Ther.* **2021**, *11*, 200–204, doi:10.22270/jddt.v11i2-S.4823.
20. Xue, C.; Lu, H.; Liu, Y.; Zhang, J.; Wang, J.; Luo, W.; Zhang, W.; Chen, J. Trans-Ferulic Acid-4-β-Glucoside Alleviates Cold-Induced Oxidative Stress and Promotes Cold Tolerance. *Int J Mol Sci* **2018**, *19*, 2321, doi:10.3390/ijms19082321.
21. Antognoni, F.; Perellino, N.C.; Crippa, S.; Dal Toso, R.; Danieli, B.; Minghetti, A.; Poli, F.; Pressi, G. Irbic Acid, a Dicaffeoylquinic Acid Derivative from Centella Asiatica Cell Cultures. *Fitoterapia* **2011**, *82*, 950–954, doi:10.1016/j.fitote.2011.05.008.
22. Magnani, C.; Isaac, V.L.B.; Correa, M.A.; Salgado, H.R.N. Caffeic Acid: A Review of Its Potential Use in Medications and Cosmetics. *Analytical Methods* **2014**, *6*, 3203–3210, doi:10.1039/C3AY41807C.
23. Pandi, A.; Kalappan, V.M. Pharmacological and Therapeutic Applications of Sinapic Acid—an Updated Review. *Molecular Biology Reports* **2021**, *48*, 3733–3745, doi:10.1007/S11033-021-06367-0/FIGURES/4.
24. Gunasekaran, S.; Venkatachalam, K.; Namasivayam, N. Anti-Inflammatory and Anticancer Effects of p-Methoxycinnamic Acid, an Active Phenylpropanoid, against 1,2-Dimethylhydrazine-Induced Rat Colon Carcinogenesis. *Molecular and Cellular Biochemistry* **2019**, *451*, 117–129, doi:10.1007/S11010-018-3398-5/FIGURES/8.
25. Chen, H.; Wang, W.; Yu, S.; Wang, H.; Tian, Z.; Zhu, S. Procyanidins and Their Therapeutic Potential against Oral Diseases. *Molecules* **2022**, *27*, doi:10.3390/MOLECULES27092932.
26. Liang, X.; Hu, C.; Liu, C.; Yu, K.; Zhang, J.; Jia, Y. Dihydrokaempferol (DHK) Ameliorates Severe Acute Pancreatitis (SAP) via Keap1/Nrf2 Pathway. *Life Sciences* **2020**, *261*, 118340, doi:10.1016/j.lfs.2020.118340.
27. Thuan, N.H.; Shrestha, A.; Trung, N.T.; Tatipamula, V.B.; Van Cuong, D.; Canh, N.X.; Van Giang, N.; Kim, T.S.; Sohng, J.K.; Dhakal, D. Advances in Biochemistry and the Biotechnological Production of Taxifolin and Its Derivatives. *Biotechnology and Applied Biochemistry* **2022**, *69*, 848–861, doi:10.1002/BAB.2156.
28. Rauf, A.; Imran, M.; Abu-Izneid, T.; Iahtisham-Ul-Haq; Patel, S.; Pan, X.; Naz, S.; Sanches Silva, A.; Saeed, F.; Rasul Suleria, H.A. Proanthocyanidins: A Comprehensive Review. *Biomedicine & Pharmacotherapy* **2019**, *116*, 108999, doi:10.1016/J.BIOPHA.2019.108999.

29. Motlhatlego, K.E.; Abdalla, M.A.; Leonard, C.M.; Eloff, J.N.; McGaw, L.J. Inhibitory Effect of Newtonia Extracts and Myricetin-3-o-Rhamnoside (Myricitrin) on Bacterial Biofilm Formation. *BMC Complement Med Ther* **2020**, *20*, 358, doi:10.1186/s12906-020-03139-4.
30. Wei, L.; Lv, S.; Huang, Q.; Wei, J.; Zhang, S.; Huang, R.; Lu, Z.; Lin, X. Pratensein Attenuates Aβ-Induced Cognitive Deficits in Rats: Enhancement of Synaptic Plasticity and Cholinergic Function. *Fitoterapia* **2015**, *101*, 208–217, doi:10.1016/j.fitote.2015.01.017.
31. Lin, Y.; Liu, P.-G.; Liang, W.-Q.; Hu, Y.-J.; Xu, P.; Zhou, J.; Pu, J.-B.; Zhang, H.-J. Luteolin-4′-O-Glucoside and Its Aglycone, Two Major Flavones of Gnaphalium Affine D. Don, Resist Hyperuricemia and Acute Gouty Arthritis Activity in Animal Models. *Phytomedicine* **2018**, *41*, 54–61, doi:10.1016/j.phymed.2018.02.002.
32. He, M.; Min, J.W.; Kong, W.L.; He, X.H.; Li, J.X.; Peng, B.W. A Review on the Pharmacological Effects of Vitexin and Isovitexin. *Fitoterapia* **2016**, *115*, 74–85, doi:10.1016/J.FITOTE.2016.09.011.
33. de Araújo Lopes, A.; Magalhães, T.R.; de Andrade Uchôa, D.E.; Silveira, E.R.; Azzolini, A.E.C.S.; Kabeya, L.M.; Lucisano-Valim, Y.M.; Vasconcelos, S.M.M.; de Barros Viana, G.S.; Leal, L.K.A.M. Afrormosin, an Isoflavonoid from Amburana Cearensis A. C. Smith, Modulates the Inflammatory Response of Stimulated Human Neutrophils. *Basic Clin Pharmacol Toxicol* **2013**, *113*, 363–369, doi:10.1111/bcpt.12106.
34. Kim, Y.W.; Zhao, R.J.; Park, S.J.; Lee, J.R.; Cho, I.J.; Yang, C.H.; Kim, S.G.; Kim, S.C. Anti-Inflammatory Effects of Liquiritigenin as a Consequence of the Inhibition of NF-KappaB-Dependent INOS and Proinflammatory Cytokines Production. *Br J Pharmacol* **2008**, *154*, 165–173, doi:10.1038/bjp.2008.79.
35. Hernández Tasco, A.J.; Ramírez Rueda, R.Y.; Alvarez, C.J.; Sartori, F.T.; Sacilotto, A.C.B.C.; Ito, I.Y.; Vichnewski, W.; Salvador, M.J. Antibacterial and Antifungal Properties of Crude Extracts and Isolated Compounds from Lychnophora Markgravii. *Natural Product Research* **2020**, *34*, 863–867, doi:10.1080/14786419.2018.1503263.
36. Pan, W.; Ikeda, K.; Takebe, M.; Yamori, Y. Genistein, Daidzein and Glycitein Inhibit Growth and DNA Synthesis of Aortic Smooth Muscle Cells from Stroke-Prone Spontaneously Hypertensive Rats. *The Journal of Nutrition* **2001**, *131*, 1154–1158, doi:10.1093/jn/131.4.1154.
37. Angeloni, S.; Navarini, L.; Khamitova, G.; Maggi, F.; Sagratini, G.; Vittori, S.; Caprioli, G. A New Analytical Method for the Simultaneous Quantification of Isoflavones and Lignans in 25 Green Coffee Samples by HPLC-MS/MS. *Food Chemistry* **2020**, *325*, 126924, doi:10.1016/j.foodchem.2020.126924.
38. Romo Vaquero, M.; García Villalba, R.; Larrosa, M.; Yáñez-Gascón, M.J.; Fromentin, E.; Flanagan, J.; Roller, M.; Tomás-Barberán, F.A.; Espín, J.C.; García-Conesa, M.-T. Bioavailability of the Major Bioactive Diterpenoids in a Rosemary Extract: Metabolic Profile in the Intestine, Liver, Plasma, and Brain of Zucker Rats. *Molecular Nutrition & Food Research* **2013**, *57*, 1834–1846, doi:10.1002/mnfr.201300052.
39. Latva-Mäenpää, H. Bioactive and Protective Polyphenolics from Roots and Stumps of Conifer Trees (Norway Spruce and Scots Pine). **2017**.
40. Shan, Y.S.; Zhang, J.; Liu, Z.; Wang, M.; Dong, Y. Developments of Combretastatin A-4 Derivatives as Anticancer Agents. *Current Medicinal Chemistry 18*, 523–538.
41. Tan, T.; Li, T.; Xiang, C.; Ouyang, Z. Toosendanin Inhibits Osteoclast Formation and Alleviate Postmenopausal Osteoporosis by Regulating the P38 Signaling Pathway. *Int Immunopharmacol* **2023**, *116*, 109745, doi:10.1016/j.intimp.2023.109745.
42. Looi, C.Y.; Arya, A.; Cheah, F.K.; Muharram, B.; Leong, K.H.; Mohamad, K.; Wong, W.F.; Rai, N.; Mustafa, M.R. Induction of Apoptosis in Human Breast Cancer Cells via Caspase Pathway by Vernodalin Isolated from Centratherum Anthelminticum (L.) Seeds. *PLoS One* **2013**, *8*, e56643, doi:10.1371/journal.pone.0056643.

43. Zhao, Q.; Yan, X.; Yue, Y.; Yue, T.; Yuan, Y. Improved Flavonoid Content in Mulberry Leaves by Solid-State Fermentation: Metabolic Profile, Activity, and Mechanism. *Innovative Food Science & Emerging Technologies* **2023**, *84*, 103308, doi:10.1016/j.ifset.2023.103308.
44. Li, S.A.; Cadelis, M.M.; Deed, R.C.; Douafer, H.; Bourguet-Kondracki, M.-L.; Michel Brunel, J.; Copp, B.R. Valorisation of the Diterpene Podocarpic Acid – Antibiotic and Antibiotic Enhancing Activities of Polyamine Conjugates. *Bioorganic & Medicinal Chemistry* **2022**, *64*, 116762, doi:10.1016/j.bmc.2022.116762.
45. Habtemariam, S. Anti-Inflammatory Therapeutic Mechanisms of Natural Products: Insight from Rosemary Diterpenes, Carnosic Acid and Carnosol. *Biomedicines* **2023**, *11*, 545, doi:10.3390/biomedicines11020545.
46. Fernández-Ochoa, Á.; Borrás-Linares, I.; Pérez-Sánchez, A.; Barrajón-Catalán, E.; González-Álvarez, I.; Arráez-Román, D.; Micol, V.; Segura-Carretero, A. Phenolic Compounds in Rosemary as Potential Source of Bioactive Compounds against Colorectal Cancer: In Situ Absorption and Metabolism Study. *Journal of Functional Foods* **2017**, *33*, 202–210, doi:10.1016/j.jff.2017.03.046.
47. Wikul, A.; Damsud, T.; Kataoka, K.; Phuwapraisirisan, P. (+)-Pinoresinol Is a Putative Hypoglycemic Agent in Defatted Sesame (Sesamum Indicum) Seeds Though Inhibiting α-Glucosidase. *Bioorganic & Medicinal Chemistry Letters* **2012**, *22*, 5215–5217.
48. Saive, M.; Genva, M.; Istasse, T.; Frederich, M.; Maes, C.; Fauconnier, M.-L. Identification of a Proanthocyanidin from Litchi Chinensis Sonn. Root with Anti-Tyrosinase and Antioxidant Activity. *Biomolecules* **2020**, *10*, 1347, doi:10.3390/biom10091347.
49. Alam, F.; Mohammadin, K.; Shafique, Z.; Amjad, S.T.; Asad, M.H.H.B. Citrus Flavonoids as Potential Therapeutic Agents: A Review. *Phytother Res* **2022**, *36*, 1417–1441, doi:10.1002/ptr.7261.
50. Sinmisola, A.; Oluwasesan, B.M.; Chukwuemeka, A.P. Blighia Sapida K.D. Koenig: A Review on Its Phytochemistry, Pharmacological and Nutritional Properties. *Journal of Ethnopharmacology* **2019**, *235*, 446–459, doi:10.1016/j.jep.2019.01.017.
51. Spaggiari, C.; Annunziato, G.; Spadini, C.; Montanaro, S.L.; Iannarelli, M.; Cabassi, C.S.; Costantino, G. Extraction and Quantification of Azelaic Acid from Different Wheat Samples (Triticum Durum Desf.) and Evaluation of Their Antimicrobial and Antioxidant Activities. *Molecules* **2023**, *28*, 2134, doi:10.3390/molecules28052134.
52. Dahibhate, N.L.; Dwivedi, P.; Kumar, K. GC–MS and UHPLC-HRMS Based Metabolite Profiling of Bruguiera Gymnorhiza Reveals Key Bioactive Compounds. *South African Journal of Botany* **2022**, *149*, 1044–1048, doi:10.1016/j.sajb.2022.02.004.
53. More, G.K.; Vervoort, J.; Steenkamp, P.A.; Prinsloo, G. Metabolomic Profile of Medicinal Plants with Anti-RVFV Activity. *Heliyon* **2022**, *8*, e08936, doi:10.1016/j.heliyon.2022.e08936.
54. Ghyselinck, N.B.; Duester, G. Retinoic Acid Signaling Pathways. *Development* **2019**, *146*, dev167502, doi:10.1242/dev.167502.
55. Rosenstock, J.; Del Prato, S. Basal Weekly Insulins: The Way of the Future! *Metabolism* **2022**, *126*, 154924, doi:10.1016/j.metabol.2021.154924.
56. Dong, H.; Wu, M.; Wang, Y.; Du, W.; He, Y.; Shi, Z. Total Syntheses and Anti-Inflammatory Activities of Syringin and Its Natural Analogues. *J Nat Prod* **2021**, *84*, 2866–2874, doi:10.1021/acs.jnatprod.1c00585.
57. He, Z. D., But, P. P. H., Chan, T. W. D., Dong, H., XU, H. X., Lau, C. P., & Sun, H.D. Antioxidative Glucosides from the Fruits of Ligustrum Lucidum. *Chemical and Pharmaceutical Bulletin* **2001**, *49*, 780–784.
58. Hai Liu, Gui-Xin Chou, Yin-Long Guo, Li-Li Ji , Jun-Ming Wang, Z.-T.W. Norclerodane Diterpenoids from Rhizomes of Dioscorea Bulbifera. *Phytochemistry* **2010**, *71*, 1174–1180.

59. Bansal, R.; Suryan, A. A Comprehensive Review on Steroidal Bioconjugates as Promising Leads in Drug Discovery. *ACS Bio and Med Chem Au* **2022**, *2*, 340–369, doi:10.1021/ACSBIOMEDCHEMAU.1C00071/ASSET/IMAGES/LARGE/BG1C00071_0024.JPEG.
60. Jiao, W.; Sang, Y.; Wang, X.; Wang, S. Metabonomics and the Gut Microbiome Analysis of the Effect of 6-Shogaol on Improving Obesity. *Food Chemistry* **2023**, *404*, 134734, doi:10.1016/j.foodchem.2022.134734.
61. Netravati; Gomez, S.; Pathrose, B.; N, M.R.; P, M.J.; Kuruvila, B. Comparative Evaluation of Anthocyanin Pigment Yield and Its Attributes from Butterfly Pea (Clitorea Ternatea L.) Flowers as Prospective Food Colorant Using Different Extraction Methods. *Future Foods* **2022**, *6*, 100199, doi:10.1016/j.fufo.2022.100199.
62. An, Y.; Luo, Q.; Han, D.; Guan, L. Abietic Acid Inhibits Acetaminophen-Induced Liver Injury by Alleviating Inflammation and Ferroptosis through Regulating Nrf2/HO-1 Axis. *Int Immunopharmacol* **2023**, *118*, 110029, doi:10.1016/j.intimp.2023.110029.
63. Simu, S.Y.; Alam, M.B.; Kim, S.Y. The Activation of Nrf2/HO-1 by 8-Epi-7-Deoxyloganic Acid Attenuates Inflammatory Symptoms through the Suppression of the MAPK/NF-KB Signaling Cascade in In Vitro and In Vivo Models. *Antioxidants* **2022**, *11*, 1765, doi:10.3390/ANTIOX11091765/S1.
64. Walker, E.H.; Pacold, M.E.; Perisic, O.; Stephens, L.; Hawkins, P.T.; Wymann, M.P.; Williams, R.L. Structural Determinants of Phosphoinositide 3-Kinase Inhibition by Wortmannin, LY294002, Quercetin, Myricetin, and Staurosporine. *Molecular Cell* **2000**, *6*, 909–919, doi:10.1016/S1097-2765(05)00089-4.
65. Berman, H.M.; Westbrook, J.; Feng, Z.; Gilliland, G.; Bhat, T.N.; Weissig, H.; Shindyalov, I.N.; Bourne, P.E. The Protein Data Bank. *Nucleic Acids Res* **2000**, *28*, 235–242.
66. Burley, S.K.; Bhikadiya, C.; Bi, C.; Bittrich, S.; Chen, L.; Crichlow, G.V.; Duarte, J.M.; Dutta, S.; Fayazi, M.; Feng, Z.; et al. RCSB Protein Data Bank: Celebrating 50 Years of the PDB with New Tools for Understanding and Visualizing Biological Macromolecules in 3D. *Protein Sci* **2022**, *31*, 187–208, doi:10.1002/pro.4213.
67. Burley, S.K.; Bhikadiya, C.; Bi, C.; Bittrich, S.; Chen, L.; Crichlow, G.V.; Christie, C.H.; Dalenberg, K.; Di Costanzo, L.; Duarte, J.M.; et al. RCSB Protein Data Bank: Powerful New Tools for Exploring 3D Structures of Biological Macromolecules for Basic and Applied Research and Education in Fundamental Biology, Biomedicine, Biotechnology, Bioengineering and Energy Sciences. *Nucleic Acids Res* **2020**, *49*, D437–D451, doi:10.1093/nar/gkaa1038.
68. Trott, O.; Olson, A.J. AutoDock Vina: Improving the Speed and Accuracy of Docking with a New Scoring Function, Efficient Optimization and Multithreading. *J Comput Chem* **2010**, *31*, 455–461, doi:10.1002/jcc.21334.
69. Eberhardt, J.; Santos-Martins, D.; Tillack, A.F.; Forli, S. AutoDock Vina 1.2.0: New Docking Methods, Expanded Force Field, and Python Bindings. *J Chem Inf Model* **2021**, *61*, 3891–3898, doi:10.1021/acs.jcim.1c00203.
70. Dallakyan, S.; Olson, A.J. Small-Molecule Library Screening by Docking with PyRx. In *Chemical Biology: Methods and Protocols*; Hempel, J.E., Williams, C.H., Hong, C.C., Eds.; Methods in Molecular Biology; Springer: New York, NY, 2015; pp. 243–250 ISBN 978-1-4939-2269-7.
71. Huang, S.-Y. Comprehensive Assessment of Flexible-Ligand Docking Algorithms: Current Effectiveness and Challenges. *Brief Bioinform* **2018**, *19*, 982–994, doi:10.1093/bib/bbx030.
72. Świechowski, M.; Godlewski, K.; Sawicki, B.; Mańdziuk, J. Monte Carlo Tree Search: A Review of Recent Modifications and Applications. *Artif Intell Rev* **2023**, *56*, 2497–2562, doi:10.1007/s10462-022-10228-y.
73. Fundamentals of Unconstrained Optimization. In *Numerical Optimization*; Nocedal, J., Wright, S.J., Eds.; Springer Series in Operations Research and Financial Engineering; Springer: New York, NY, 2006; pp. 10–29 ISBN 978-0-387-40065-5.

74. Interior-Point Methods for Nonlinear Programming. In *Numerical Optimization*; Nocedal, J., Wright, S.J., Eds.; Springer Series in Operations Research and Financial Engineering; Springer: New York, NY, 2006; pp. 563–597 ISBN 978-0-387-40065-5.
75. Wang, R.; Lai, L.; Wang, S. Further Development and Validation of Empirical Scoring Functions for Structure-Based Binding Affinity Prediction. *J Comput Aided Mol Des* **2002**, *16*, 11–26, doi:10.1023/a:1016357811882.
76. Wang, R.; Fang, X.; Lu, Y.; Wang, S. The PDBbind Database: Collection of Binding Affinities for Protein-Ligand Complexes with Known Three-Dimensional Structures. *J Med Chem* **2004**, *47*, 2977–2980, doi:10.1021/jm030580l.
77. Wang, R.; Fang, X.; Lu, Y.; Yang, C.-Y.; Wang, S. The PDBbind Database: Methodologies and Updates. *J Med Chem* **2005**, *48*, 4111–4119, doi:10.1021/jm048957q.
78. Grosdidier, A.; Zoete, V.; Michielin, O. Fast Docking Using the CHARMM Force Field with EADock DSS. *J Comput Chem* **2011**, *32*, 2149–2159, doi:10.1002/jcc.21797.
79. Grosdidier, A.; Zoete, V.; Michielin, O. SwissDock, a Protein-Small Molecule Docking Web Service Based on EADock DSS. *Nucleic Acids Res* **2011**, *39*, W270–W277, doi:10.1093/nar/gkr366.
80. Grosdidier, A.; Zoete, V.; Michielin, O. Blind docking of 260 protein–ligand complexes with EADock 2.0. *Journal of Computational Chemistry* **2009**, *30*, 2021–2030, doi:10.1002/jcc.21202.
81. Brooks, B.R.; Brooks III, C.L.; Mackerell Jr., A.D.; Nilsson, L.; Petrella, R.J.; Roux, B.; Won, Y.; Archontis, G.; Bartels, C.; Boresch, S.; et al. CHARMM: The Biomolecular Simulation Program. *Journal of Computational Chemistry* **2009**, *30*, 1545–1614, doi:10.1002/jcc.21287.
